# Transcriptome analysis of grain-filling caryopses reveals involvement of multiple regulatory pathways in chalky grain formation in rice

**DOI:** 10.1186/1471-2164-11-730

**Published:** 2010-12-30

**Authors:** Xiaolu Liu, Tao Guo, Xiangyuan Wan, Haiyang Wang, Mingzhu Zhu, Aili Li, Ning Su, Yingyue Shen, Bigang Mao, Huqu Zhai, Long Mao, Jianmin Wan

**Affiliations:** 1 Institute of Crop Sciences, Chinese Academy of Agriculture Sciences, Beijing, 100081, China; 2 State Key Laboratory of Crop Genetics and Germplasm Enhancement/Jiangsu Plant Gene Engineering Research Center, Nanjing Agricultural University, Nanjing 210095, Jiangsu, China; 3 Chinese Academy of Agriculture Sciences, Beijing, 100081, China

## Abstract

**Background:**

Grain endosperm chalkiness of rice is a varietal characteristic that negatively affects not only the appearance and milling properties but also the cooking texture and palatability of cooked rice. However, grain chalkiness is a complex quantitative genetic trait and the molecular mechanisms underlying its formation are poorly understood.

**Results:**

A near-isogenic line CSSL50-1 with high chalkiness was compared with its normal parental line Asominori for grain endosperm chalkiness. Physico-biochemical analyses of ripened grains showed that, compared with Asominori, CSSL50-1 contains higher levels of amylose and 8 DP (degree of polymerization) short-chain amylopectin, but lower medium length 12 DP amylopectin. Transcriptome analysis of 15 DAF (day after flowering) caryopses of the isogenic lines identified 623 differential expressed genes (*P *< 0.01), among which 324 genes are up-regulated and 299 down-regulated. These genes were classified into 18 major categories, with 65.3% of them belong to six major functional groups: signal transduction, cell rescue/defense, transcription, protein degradation, carbohydrate metabolism and redox homeostasis. Detailed pathway dissection demonstrated that genes involved in sucrose and starch synthesis are up-regulated, whereas those involved in non-starch polysaccharides are down regulated. Several genes involved in oxidoreductive homeostasis were found to have higher expression levels in CSSL50-1 as well, suggesting potential roles of ROS in grain chalkiness formation.

**Conclusion:**

Extensive gene expression changes were detected during rice grain chalkiness formation. Over half of these differentially expressed genes are implicated in several important categories of genes, including signal transduction, transcription, carbohydrate metabolism and redox homeostasis, suggesting that chalkiness formation involves multiple metabolic and regulatory pathways.

## Background

Endosperm chalkiness is a varietal characteristic that negatively affects not only the appearance and milling properties but also the cooking texture and palatability of cooked rice [1]. Chalky grains have a lower density of starch granules compared to vitreous ones, and are therefore more prone to breakage during milling [2]. In many rice-producing areas, high chalkiness is a major concern that decreases grain quality. In China, many early-season indica and japonica varieties are of high grain endosperm chalkiness and their market values are seriously affected [1]. Therefore, one of the goals in rice breeding is to reduce chalkiness in rice varieties.

In rice grains, starch is the predominant storage substance that account for over 80% of the total dry mass. Starch in rice endosperm is composed of relatively unbranched amylose (linear α-1, 4-polyglucans) and highly branched amylopectin (α-1, 6-branched polyglucans). Recent work showed that multiple factors contribute to the formation of grain chalkiness, including starch synthesis, starch granule structure and arrangement [3-5]. For example, mutations in the *Wx *(*waxy*) gene (encoding grandule-bound sucrose synthase, or GBSS) and its regulator *DULL *cause low amylose content (≤ 2%) and hence whole opaque endosperm [6,7]. The *amylose-extender *mutant has reduced activity of branching enzyme II (BEIIb), causing alteration in the fine structure of grain amylopectin [8]. The *flo-2 *floury endosperm mutant harbors mutations affecting rice branching enzyme I (RBEI) activity [9]. The *floury endosperm-4 *mutant and the *sugary-1 *mutant are defective in pyruvate orthophosphate dikinase (PPDK) and debranching enzymes (DBE) activity respectively [4,10].

The formation of grain chalkiness can also be influenced by various external stresses during the grain-filling stage. Temperatures higher than 26°C, for example, could easily cause chalky appearance and a reduction in grain weight [11]. Microscopic observation showed that, compared with the translucent portion of rice endosperm that ripened under normal temperature which were filled with densely packed and polygonal granules, the chalky portion of high temperature-ripened grains were loosely packed with elliptical-shaped starch granules containing air spaces which caused random light reflection and hence chalky appearance [5,12-14]. These observations demonstrated that environmental stresses represent another major cause for grain chalkiness in rice. Furthermore, imaging on endosperm amyloplast development of various japonica and indica rice lines indicated that starch synthesis in the rice grain may involve complicated genetic networks [4,15]. Previous studies have detected many major quantitative trait loci (QTLs) that may underlie chalkiness in rice [16-19], however; only few QTLs have been isolated and functionally analyzed [20]. Thus, the molecular mechanisms underlying the formation of rice grain endosperm chalkiness still remain poorly understood.

In this study, we performed a comparative transcriptome analysis of the caryopses of a near-isogenic line CSSL50-1 (with high chalkiness) and its low chalkiness parental line Asominori. Corroborated with the phenotypic and physico-biochemical observations, our genome-wide transcription analysis supports the notion that rice grain endosperm development is controlled by a delicate, but complex genetic network. Notably, several pathways related to signal transduction, cell rescue/defense, transcription, protein degradation, carbohydrate metabolism and redox homeostasis were found to be predominant among the differentially expressed genes.

## Results

### Phenotypic and physiochemical properties of Asominori and CSSL50-1 grains

CSSL50-1 is derived from the near-isogenic line CSSL50 with a small substituted segment of chromosome 8 from the original donor IR24 in the largely Asominori background [21]. CSSL50-1 displays high chalkiness under normal field conditions whereas its parental line Asominori has normal grains. Therefore, CSSL50-1 represents an ideal genetic material with relatively stabilized genetic background suitable for exploring the molecular mechanism of chalkiness formation.

**Figure 1:**
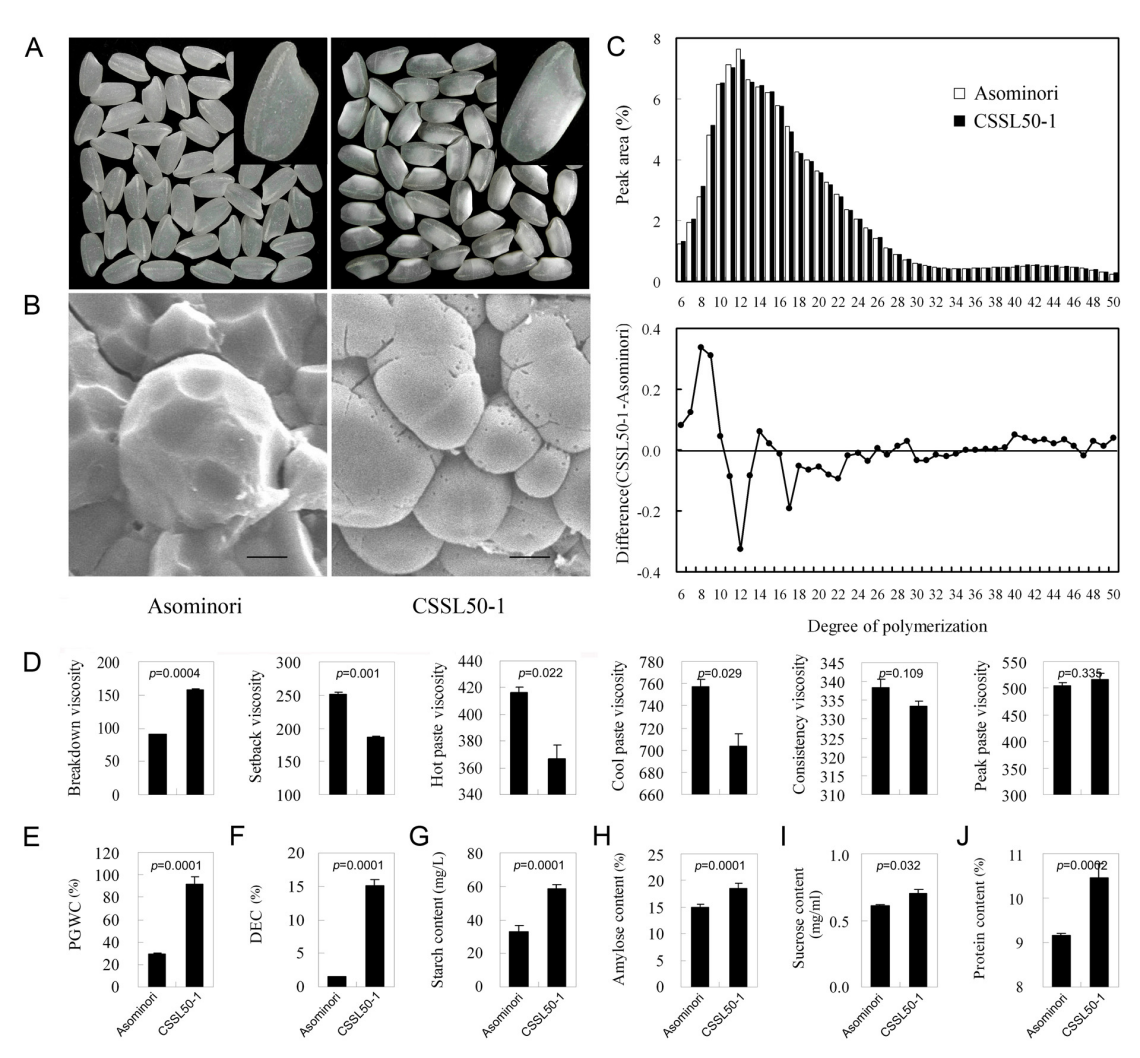
**Starch properties of Asominori and CSSL50-1**. (A) Rice grains of Asominori (left) and CSSL50-1 (right); (B) Scanning electron microscopy of starch granule transverse sections of Asominori (left) and CSSL50-1 (right); (C) Chain-length profile of Asominori and CSSL50-1 and differences in the chain-length distribution of amylopectin; (D) RVA profile, Rapid Visco Analyzer profile (E) PGWC, Percentage of grain with chalkiness; (F) DEC, Degree of endosperm chalkiness; (G) StC, Starch content; (H) AC, Amylose content; (I) SuC, sucrose content; (J) PC, protein content. Bars = 3.0 μm.

CSSL50-1 grains display higher chalkiness with less translucence, when compared with its parental line Asominori (Figure 1A). Scanning electron microscopy showed that the chalky endosperm is comprised of round and loosely packed starch granules with large air spaces, in contrast to the translucent Asominori grains that are filled with densely packed granules (Figure 1B). CSSL50-1 grains have a higher content of short-chain amylopectins (8-9 DP, or degree of polymerization), but less medium (12 DP) or long (17 DP) amylopectin chains (Figure 1C). This observation is consistent with the Rapid Visco Analyzer (RVA) profile which provides a comprehensive evaluation of the grain quality (Figure 1D). The relatively lower ratio of medium or long chain amylopectin in CSSL50-1 is correlated with the higher breakdown frequency of its starch granule when heated, indicating that the importance of the fine structure of amylopectin in normal starch granule appearance and degree of grain chalkiness [22]. Overall, CSSL50-1 has a higher percentage of grain with chalkiness (PGWC), chalkiness percentages, degree of endosperm chalkiness (DEC), starch content, amylose content, sucrose content and protein content when compared with Asominori (Figure 1E-J). These results collectively indicate that the occurrence of grain chalkiness is associated with changes in starch granule shape, amylopectin chain-length profiles, amylose and protein content, and RVA profile characteristics.

### Increased grain-filling rate and enhanced activities of starch enzymes in CSSL50-1

**Figure 2:**
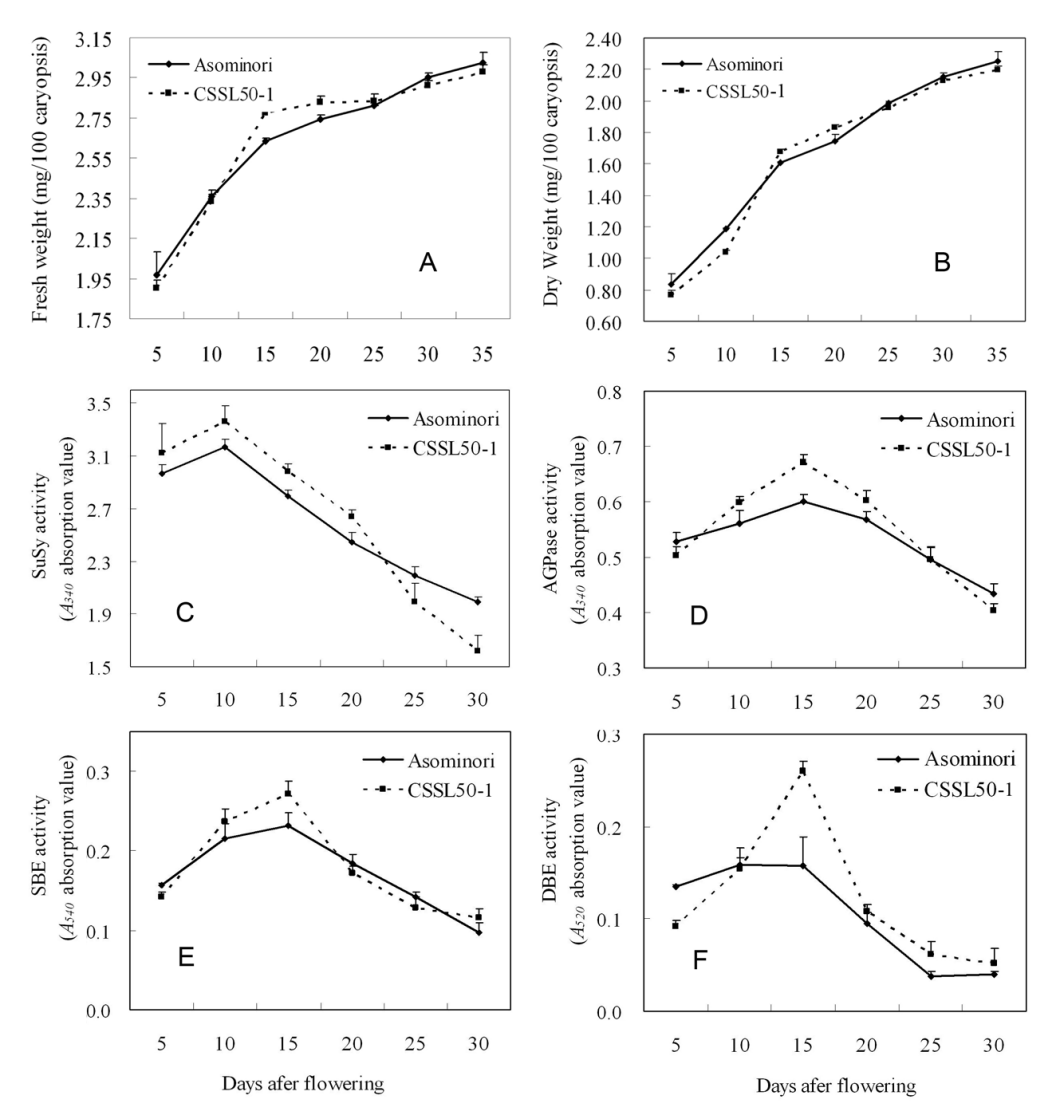
**Comparison of grain-filling rates and activities of starch synthesis enzymes between CSSL50-1 and Asominori**. (A) Fresh weight of rice grains; (B) Dry weight of rice grains; (C) Sucrose synthase (SuSy); (D) ADP-glucose pyrophosphorylase (AGPase); (E) Starch branching enzyme (SBE); (F) Starch debranching enzyme (DBE).

The observation that CSSL50-1 grains are high in short-chain amylopectin, but low in medium and long ones suggest that the grain-filling rate at early stage of grain development may be faster in CSSL50-1 than in Asominori. We thus measured the fresh and dry grain weight at several grain-filling stages (5, 10, 15, 20, 25, 30 and 35 day after flowering, or DAF) for CSSL50-1 and Asominori. The results indeed showed that grain-filling rate at 15, 20, and 25 DAF is faster in CSSL50-1 than that in Asominori (Figure 2A &2B). In contrast, Asominori exhibits a smooth and steady grain-filling course. These results suggest that the faster grain-filling pace before 15 DAF in CSSL50-1 could be an important contributing factor for the formation of chalkiness at the later stage of endosperm development. This notion is consistent with a previous study showing that a steady grain-filling rate is required for prevention of chalkiness in rice endosperm [5]. Unexpectedly, no significant changes were detected in photosynthesis efficiency in CSSL50-1 rice leaves during 10-15 DAF of the grain filling stage, suggesting that photosynthesis efficiency is not tightly linked with chalkiness formation in rice grains (Additional file 1, Figure S1).

Since grains of CSSL50-1 contain higher starch, amylose and sucrose contents compared with Asominori, we speculated that enzymes involved in starch synthesis might be more robust in CSSL50-1 than in Asominori. To confirm this, the enzymatic activities of four major enzymes involved in grain starch synthesis were measured during the first 30 days after flowering. Similar patterns were observed for SuSy in CSSL50-1 and Asominori, namely, its highest activity was detected at 10 DAF, and its lowest activity detected at 30 DAF (Figure 2C). However, SuSy activity in CSSL50-1 was higher than that in Asominori at the 15 and 20 DAF. Similarly, at 15 DAF, AGPase, SBE and DBE activities were significantly higher in CSSL50-1 compared to those in Asominori (Figure 2D-F). Additionally, enzyme activities of SuSy at 30 DAF and DBE at 5 DAF were found to be lower in CSSL50-1 than those in Asominori (Figure 2C). These results indicate that 15 DAF is a critical time point for grain filling when many enzymes involved in starch synthesis exhibit maximum activities. We therefore used RNAs extracted from 15 DAF endosperms for subsequent microarray analysis.

### Transcriptome analysis of 15 DAF caryopses of CSSL50-1 and Asominori

**Figure 3:**
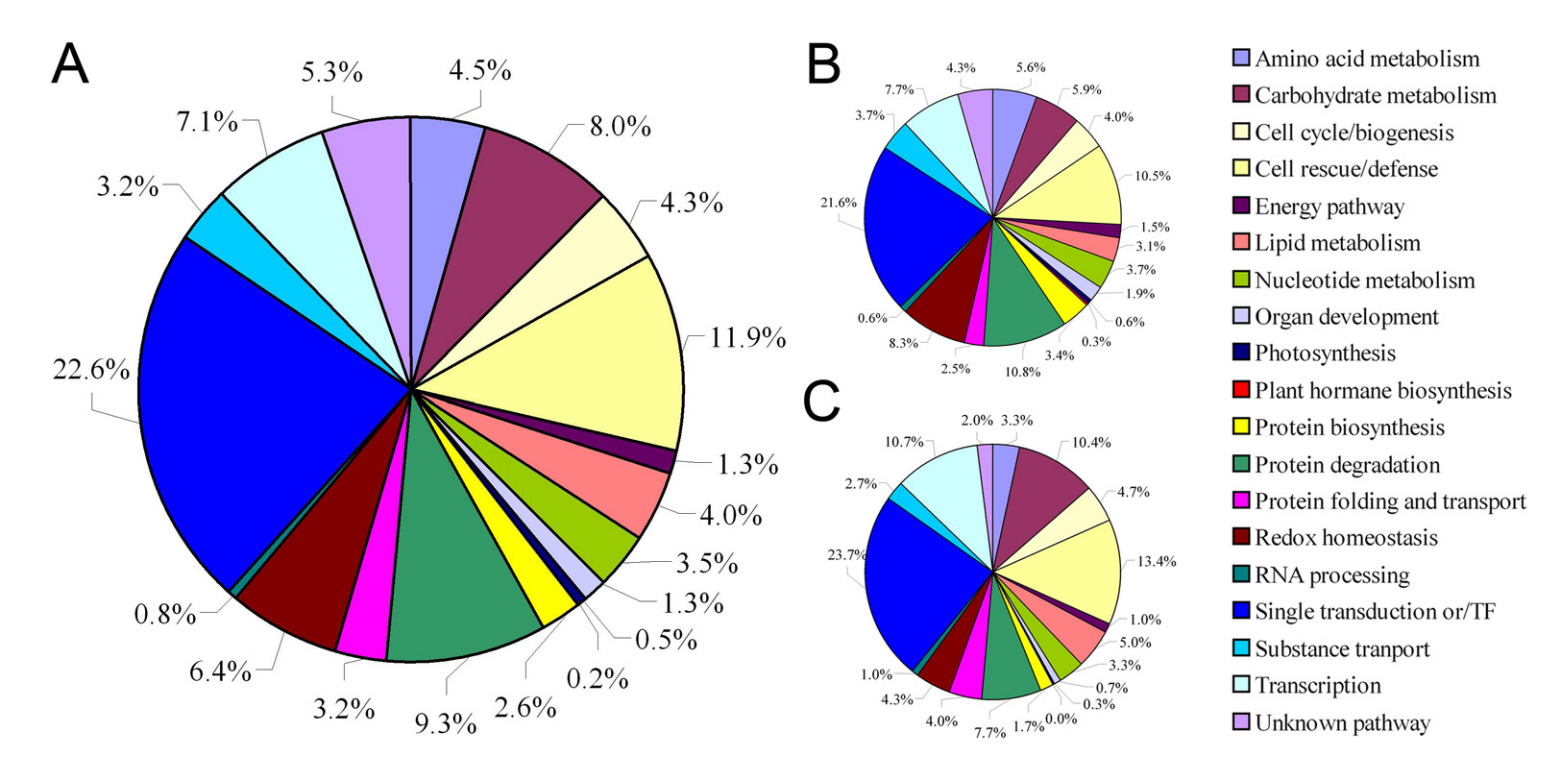
**Functional classification and distribution of 623 differentially expressed genes**. (A) Total differentially expressed genes (623); (B) Genes up-regulated (324); (C) Genes down-regulated (299).

To investigate the underlying molecular basis for chalky endosperm formation, we used Affymetrix GeneChips for a global transcriptome profiling analysis (Additional file 2, Figure S2). A total of 2295 transcripts were found to be differentially expressed between CSSL50-1 and Asominori with FDR ≤5% using the Significance Analysis of Microarray (SAM) software. Among these, 798 transcripts differ more than 1.5 fold and 193 transcripts differ more than 2.0 fold between Asominori and CSSL50-1. Fisher's exact test showed that 10 functional terms in Biological Process and two Molecular Function terms were significantly enriched among these genes (Table 1). Interesting categories that may be involved in rice endosperm development were carbohydrate metabolism, response to stress, transcription, hydrolase activity, and oxidoreductase activity. Gene Ontology (GO) annotation of the 193 transcripts with ≥2 fold change was listed in Additional file 3 (Table S1). Genes in carbohydrate metabolism includes glucose-6-phosphate isomerase, alpha-amylase, and glycosyl hydrolases family 1, 16, and 17 proteins. Genes of the oxidoreductase activity group includes L-ascorbate peroxidase 3, glutathione S-transferase, peroxidase 64, and monodehydroascorbase reductase that are known to be involved in redox homeostasis. Transcription factors include genes encoding one Myb-like DNA-binding domain containing protein, two AP2 domain proteins, one homeobox domain protein and one GAF domain containing protein. The functions of a large number of genes were classified as *primary metabolic process*, including genes encoding a U-box domain containing protein and an ubiquitin carboxy-terminal hydrolase that may be involved in protein degradation, several protein kinases for signaling transduction, two leucine-rich repeat family proteins that may be associated with defense response. These observations suggest that intricate a gene network may underlie the proper development of rice grain endosperms. To further improve the stringency, we applied one-way ANOVA analysis on the differentially expressed genes identified by SAM. This analysis identified 623 statistically differentially expression genes (*P *< 0.01, Additional file 4, Table S2). Following the functional categories given by GO and the bioinformatics tool JAFA http://jafa.burnham.org, we manually classified these genes into 18 major categories, including signal transduction, cell rescue/defense, transcription, protein degradation, carbohydrate metabolism, redox homeostasis, amino acid metabolism, cell cycle/biogenesis, lipid metabolism, nucleotide metabolism, substance transport, protein folding and transport, protein biosynthesis, organ development, energy pathway, RNA processing, photosynthesis, and plant hormone biosynthesis (Figure 3). Consistent with what we observed in GO analysis (Additional file 4, Table S2), 65.3% of these genes belong to the first six functional groups (signal transduction, cell rescue/defense, transcription, protein degradation, carbohydrate metabolism and redox homeostasis), suggesting that the occurrence of endosperm chalkiness in rice might be closely related to these functional and regulatory pathways. In addition, only three genes associated with photosynthesis were differentially expressed between Asominori and CSSL50-1 (Additional file 4, Table S2), implying that photosynthesis efficiency may not play a significant role in the formation of chalkiness in rice.

**Table 1:** Functional classification of differentially expressed genes associated with enriched GO terms1

GO components		Gene number^3^	*P *value^2^	Depth
**A. Biological process**				
lipid transport	GO:0006869	7	3.61E-07	3
lipid localization	GO:0010876	7	3.61E-07	3
response to stress	GO:0006950	12	2.33E-05	2
carbohydrate metabolic process	GO:0005975	11	6.38E-04	3
macromolecule localization	GO:0033036	7	1.91E-03	2
regulation of transcription, DNA-dependent	GO:0006355	8	4.07E-03	5
regulation of RNA metabolic process	GO:0051252	8	4.17E-03	4
transcription, DNA-dependent	GO:0006351	8	5.69E-03	5
RNA biosynthetic process	GO:0032774	8	5.69E-03	4
primary metabolic process	GO:0044238	39	6.51E-03	2
**B. Molecular function**				
hydrolase activity, hydrolyzing O-glycosyl compounds	GO:0004553	7	8.41E-04	4
oxidoreductase activity	GO:0016491	20	4.41E-03	2

^1^Differentially expressed genes with fold change of > = 2 are classified here.

^2^*P *values were calculated using Fisher's exact test.

^3^Only groups with more than 5 genes/transcripts were listed here.

### Enhanced sucrose and starch synthesis vs. disrupted cellulose, hemicellulose and pectin metabolism in CSSL50-1

Physio-biochemical analysis of chalky rice endosperm indicated that the change in starch composition is a major difference between chalky and non-chalky rice grains (Figure 1 & Figure 2). GO analysis also showed that genes associated with carbohydrate metabolism are significantly represented among the differentially expressed transcripts. As shown in Additional file 4 (Table S2), more than 50 genes are annotated to be associated with carbohydrate metabolism. Of particular interest were several key genes that are known to be directly involved in the synthesis of starch and cell-wall related polysaccharides (eg. sucrose phosphatase, sucrose phosphate synthase, glucose-6-phosphate isomerase, glycosyltransferae family 5, starch debranching enzyme, α-amylase, cellulose synthesis and α-D-xylosidase).

**Figure 4:**
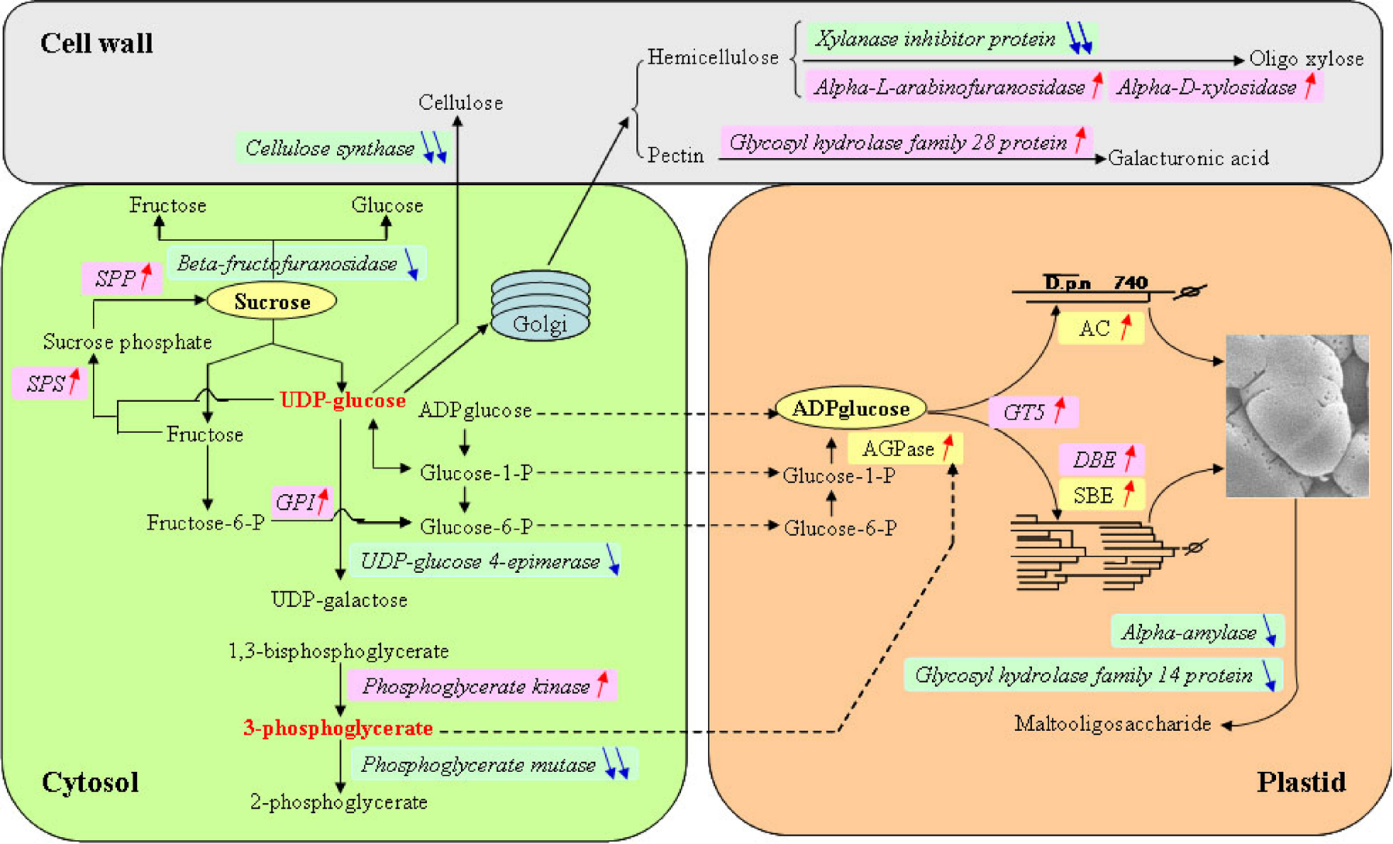
**Overlays of differentially expressed genes onto starch and non-starch polysaccharide metabolism pathways**. Up arrows designate up-regulation of the genes and down arrows down-regulated genes. The number of arrows indicates the number of genes. AC, amylose content; AGPase, ADP-glucose pyrophosphorylase; DBE, starch debranching enzyme; GPI, glucose-6-phosphate isomerase; GT5, glycosyltransferase family 5 protein; SBE, starch branching enzyme; SPP, sucrose phosphatase; SPS, sucrose phosphate synthase.

A closer examination of these carbohydrate metabolism genes revealed that the differentially expressed genes in CSSL50-1 were in favor of enhancing sucrose, amylose, and amylopectin synthesis. As shown in Figure 4, two genes, sucrose phosphatase (SPP) and sucrose phosphate synthase (SPS) that directly catalyze sucrose synthesis, are up-regulated, whereas the enzyme β-fructofuranosidase that catalyzes the hydrolysis of sucrose to glucose and fructose is down-regulated. The potentially accumulated sucrose (Figure 1I), catalyzed by the reversible enzymatic activity of sucrose synthase (SuSy) (Figure 2C), may increase the concentration of UDP-glucose, which can be converted into glucose-1-phosphate and subsequently converted into ADP-glucose for starch synthesis. Microarray data also revealed several additional enzymes that are up-regulated in CSSL50-1 for the accumulation of ADP-glucose: (1) up-regulation of glucose-6-phosphate isomerase to promote fructose-6-phosphate to glucose-6-phosphate conversion; (2) down-regulation of UDP-glucose 4-epimerase to reduce conversion of UDP-glucose to UDP-galactose; (3) up-regulation of phosphoglycerate kinase and down-regulation of phosphoglycerate mutase for the accumulation of 3-phosphoglycerate, an activator for ADP-glucose pyrophosphorylase that converts glucose-1-phosphate to ADP-glucose [23]. In addition, several enzymes are regulated to promote starch component synthesis: (1) up-regulation of a gene encoding a protein of the glycosyltransferae family 5 for increased amylose content; (2) up-regulation of starch branching enzyme and starch debranching enzyme that promote amylopectin elongation and branching; (3) down-regulation of α-amylase and glycosyl hydrolase family 14 protein to reduce starch degradation to malto-oligosaccharide. Therefore, the enhanced enzymatic activities for sucrose and starch synthesis correlate with the high content of sucrose, amylose, and starch in CSSL50-1 (Figure 1 & Figure 2)

In CSSL50-1, the enhancement of sucrose and starch (Figure 1I) seems to be accompanied by a metabolic disorder of cell wall related polysaccharides. First, two cellulose synthase genes were down-regulated which may reduce cellulose synthesis; Second, up-regulation of α-L-arabinofuranosidase and α-D-xylosidase and down-regulation of an xylanase inhibitor protein may promote hydrolysis of hemicellulose [24]. These observations seem to suggest that the enhancement of sucrose and starch synthesis is at the cost of cell wall related non-storage polysaccharides in CSSL50-1. Such carbohydrate metabolism disorders may significantly contribute to the endosperm chalkiness during grain ripening.

### Increased expression of redox genes and a higher level ROS homeostasis in CSSL50-1

Previous studies showed that rice grains develop chalkiness under adverse environmental conditions such as high temperatures [5]. GO analysis also indicated significant enrichment in *oxidoreductase activity *in Molecular Function (Table 1 & Additional file 3, Table S1). About 40 genes fell in the category of *redox homeostasis *in our manual classification of differentially expressed genes (Additional file 4 Table S2). Since reactive oxygen species (ROS) are well known to be involved in various stress responses, we first measured the concentration of H_2_O_2_, a common ROS, in CSSL50-1 and Asominori. The results showed that the 15 DAF grains of CSSL50-1 contained much higher H_2_O_2 _concentration (617 umol/g fresh weight) than that in Asominori (451.3 umol/g fresh weight) (*P *< 0.01). Such an imbalance in ROS concentrations and its consequence may contribute to the development of chalkiness in grain endosperm at later developmental stages.

**Figure 5:**
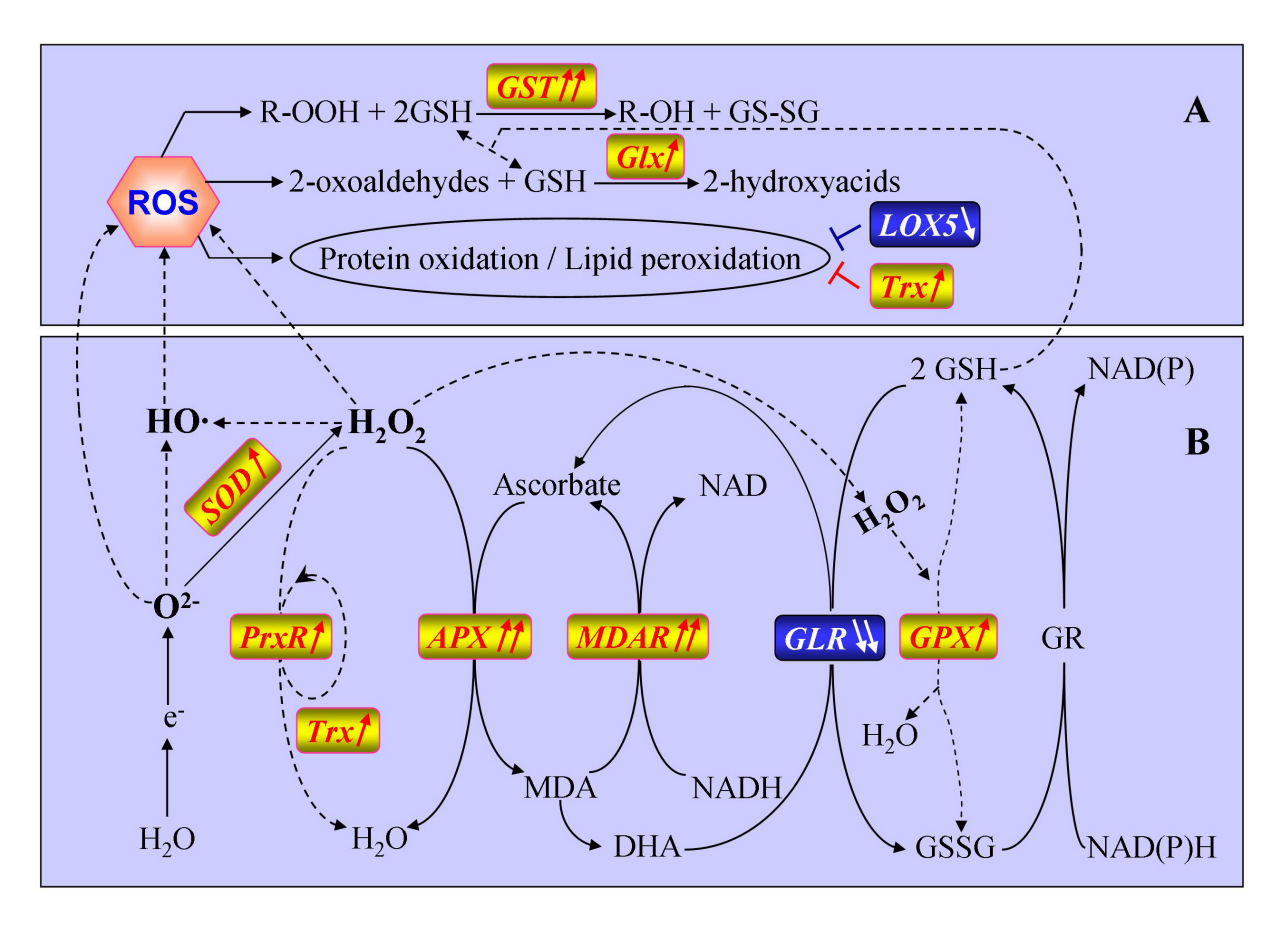
**Differentially expressed genes associated with redox homeostasis networks in plants**. Up arrows designate up-regulation of the genes and down arrows down-regulated genes. The number of arrows indicates the number of genes. APX, ascorbate peroxidase; DHA, dehydroascrobate; GLR, glutaredoxin; Glx, glyoxalase; GPX, glutathione peroxidase; GR, glutathione reductase; GSH, reduced glutathione; GSSG, oxidized glutathione; GST, glutathione-S-transferase; LOX5, lipoxygenase-5; MDA, monodehydroascorbate; MDAR, MDA reductase; PrxR, peroxiredoxin; ROS, reactive oxygen species; SOD, superoxide dismutase; Trx, thioredoxin.

Our microarray analysis revealed that the major enzyme responsible for converting free radicals to H_2_O_2_, the superoxide dismutase (SOD) gene, is up-regulated 1.67 fold in CSSL50-1. Genes encoding five other enzymes involved in H_2_O_2 _clearance, such as peroxiredoxin (PrxR), ascorbate peroxidase (APX), monodehydroascorbate reductase (MDAR), and peroxiredoxin (PrxR), are also up-regulated, except for two glutaredoxin genes (Figure 5B). Additionally, four genes involved in oxidized product clearance are regulated in favor of maintaining a homeostasis of these deleterious molecules in CSSL50-1. These are glutathione-S-transferase (GST), glyoxalase (Glx), lipoxygenase-5 (LOX5), and thioredoxin (Trx) (Figure 5A). These enzymes function to remove oxidized proteins and lipids [25,26]. Together, these observations suggest a close correlation between ROS homeostasis and grain chalkiness.

### A delicate but complicated gene network may underlie chalky rice grain formation

**Figure 6:**
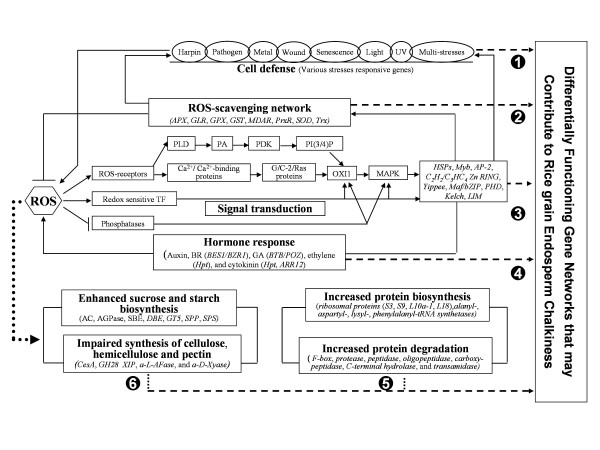
**An overview of the major gene networks closely associated with rice grain endosperm chalkiness**. AC, amylose content; a-D-Xyase, Alpha-D-xylosidase; AGPase, ADP-glucose pyrophosphorylase; a-L-AFase, Alpha-L-arabinofuranosidase; AP-2, APETALA2 domain-containing protein; APX, ascorbate peroxidase; ARR, arabidopsis thaliana response regulator; BES1/BZR1, BES1/BZR1 homolog protein; BR, brassinosteroid; BTB/POZ, BTB/POZ domain protein; C2H2/C3HC4 Zn RING, C2H2/C3HC4-type zinc finger proteins; CesA, cellulose synthase; DBE, starch debranching enzyme; GA, gibberellin; GH28, Glycosyl hydrolase family 28 protein; GLR, glutaredoxin; GPX, glutathione peroxidase; GST, glutathione-S-transferase; GT5, glycosyltransferase family 5 protein; Hpt, Hpt domain protein; HSP, heat shock protein; LIM, LIM domain protein; Maf/bZIP, Maf family protein; MAPK, mitogen-activated protein kinase; MDAR, monodehydroascorbate reductase; Myb, Myb transcription factor; OXI1, serine/threonine protein phosphatase; PA, phosphatidic acid; PDK, phosphoinositide-dependent kinase; PHD, PHD finger transcription factor; PI(3/4)P, phosphatidylinositol-4-phosphate; PLD, phospholipase D; PrxR, peroxiredoxin; ROS, reactive oxygen species; SBE, starch branching enzyme; SOD, superoxide dismutase; SPP, sucrose phosphatase; SPS, sucrose phosphate synthase; TF, transcription factor; Trx, thioredoxin; XIP, Xylanase inhibitor protein.

Additional file 4 (Table S2) lists the genes that were differentially expressed between CSSL50-1 and Asominori. In addition to those involved in starch and redox homeostasis that have been detailed above, genes involved in additional biological processes such as cell rescue/defense, hormone response, and protein biosynthesis and degradation are also differentially expressed between CSSL50-1 and Asominori. It is noteworthy that most genes involved in these pathways did not change significantly in terms of fold changes. Such a result, however, is similar to a previous cDNA array study of grain chalkiness under high temperature [5]. The subtle change in gene expression and the significant consequence in endosperm chalkiness formation seems to suggest that rice grain filling is a fine-tuned process which can be easily affected by genetic variations as well as fluctuations in environmental conditions. We therefore depicted a possible gene network according to the microarray data. As shown in Figure 6, in addition to the enhanced carbohydrate metabolisms for starch and suppressed non-starch polysaccharides and an elevated ROS homeostasis, changes of gene expression levels in four additional pathways may also play roles in chalkiness formation of rice grains: (1) genes that are known to be involved in biotic and abiotic stress responses, encoding those such as the NB-ARC domain containing proteins, the leucine rich repeat family proteins and harpin-induced proteins, as well as heavy metal binding proteins and proteins involved in wound, senescence, light, UV and other stress responses; (2) Genes involved in ROS signaling such as phospholipase D, phosphatases, Ca^2+^/Ca^2+^-binding protein, G-proteins, and Ras proteins; (3) Hormone biosynthesis and signaling related genes, such as auxin, BR (BES1/BZR1), GA (BTB/POZ), ethylene (Hpt) and cytokinin (Hpt and ARR12); (4) Genes involved in protein synthesis, such as those encoding ribosomal S3, S9, S11, L10a-1 and L18 subunits and alanyl-, aspartyl-, lysyl-, phenylalanyl-tRNA synthetases and degradation, such as those encoding F-box, protease, peptidase, oligopeptidase, carboxy-peptidase, C-terminal hydrolase and transamidase. Therefore, the formation of grain chalkiness likely involves alterations in multiple biological processes and multiple genetic pathways.

**Figure 7:**
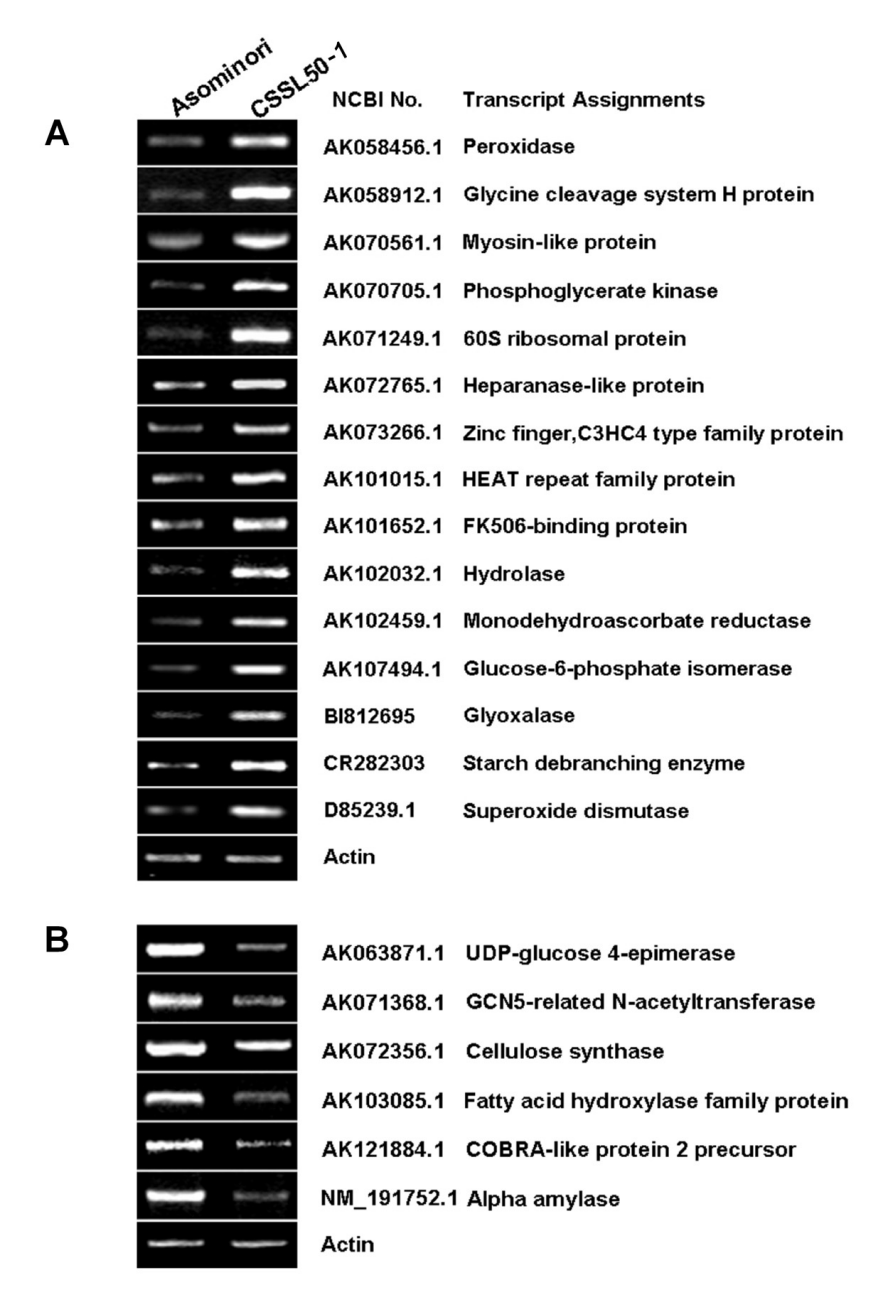
**RT-PCR confirmation of the microarray data**. Twenty-one differentially expressed genes were randomly selected for RT-PCR analysis. (A) 15 up-regulated genes; (B) 6 down-regulated genes. Actin was used as the control.

For confirmation, 21 transcripts were randomly chosen for semi-quantitative RT-PCR analysis. The RT-PCR results correlate well with the microarray data, thus validating our microarray data (Figure 7).

## Discussion

### CSSL50-1 is an ideal material for exploring the molecular basis of rice grain chalkiness

Grain endosperm chalkiness is a complex quantitative genetic trait and is controlled by multiple factors [19]. Previous studies showed that there are as many as 42 QTLs that may contribute to the percentages of grains with chalkiness (PGWC) and degrees of endosperm chalkiness. These genes spread among 10 rice chromosomes as being located using seven different genetic populations [16,17,19,27-30]. In addition to genetic factors, rice grains are also sensitive to various environmental stresses and will easily form chalky endosperms under adverse conditions. Changes in temperature and light, for example, are most effective at the milky stage of grain filling [5,11,31]. Meanwhile, conditions of water and nutrient in the field also play important roles on grain chalkiness [32,33].

Despite its economic importance, only few genes have been functionally identified to be associated with endosperm chalkiness. This analysis, together with a previous transcriptome analysis on chalkiness formation under higher temperature [5], has identified a set of differentially expressed genes that may contribute to endosperm chalkiness. The identified candidate genes may serve as excellent starting materials for dissecting the pathway controlling rice endosperm development at the molecular level. Notably, most of these genes identified in this study belong to six major categories including cell rescue/defense, free radical clearance and redox homeostasis, signal transduction, hormone response, protein biosynthesis and degradation, and carbohydrate metabolism. Our data also showed that, similar to the effect of high temperature, expression of numerous genes was affected under this genetic background, but surprisingly, quite a few of these genes were oppositely regulated in CSSL50-1 when compared with the effect under the high temperature conditions. Thus, the use of a genetically stabilized line for endosperm chalkiness study is complementary to the previous physical stress-based studies and should provide novel information regarding the molecular mechanism for chalky endosperm formation in rice.

### Enhanced starch synthesis causes imbalanced starch composition in CSSL50-1

Previously, chalky grains were found to have lower starch content [5]. For CSSL50-1, its grains contain higher percentage of sucrose, amylose, starch, and even protein content when compared with the normal rice cultivar Asominori. Enzymes, such as SuSy, AGPase, SBE and DBE, exhibit higher activities at 15 DAF in CSSL50-1, correlating with the higher expression levels of corresponding genes that were detected in our microarray data. Since the shape and the arrangement of starch granule are closely related to endosperm chalkiness in rice [3,4,34], it is reasonable to conclude that a coordinated and balanced action of starch synthetic enzymes are critical to the prevention of chalky endosperm formation.

Endosperm development is a process of proper starch composition and accumulation [35,36]. Gradual and smooth grain filling pace is required to form normal, translucent grains as seen in Asominori. CSSL50-1 has a higher grain-filling rate which may give insufficient time for long chain amylopectin to be synthesized, resulting in a relative higher percentage of short chain amylopectin (8-9 DP) when compared with Asominori. Consistent with our results, the decrease of 10-14 DP amylopectin was also observed when rice grains ripened under high temperatures [5]. This is in line with our findings that both gene expression and the enzymatic activity of DBE are increased in CSSL50-1. Interestingly, the synthesis of non-starch polysaccharides appears to be significantly retarded because of the down regulation of related genes such as cellulose synthase and the up-regulation of genes for degrading hemicellulose and pectin. Therefore, the synthesis of cell wall related sugar might be sacrificed in CSSL50-1. In light of the importance of normal synthesis of starch and related polysaccharides, it is very likely that disorders in the enzyme activity and the expression of genes responsible for these events are among the major causes for endosperm chalkiness in CSSL50-1.

### Potential roles of ROS in rice grain chalkiness formation

Reactive oxygen species (ROS) are partially reduced forms of atmospheric oxygen (O_2_). They typically result from the excitation of O_2 _to form singlet oxygen (O^-^) or from the transfer of one, two or three electrons to O_2 _to form, respectively, a superoxide radical (O_2_^-^), hydrogen peroxide (H_2_O_2_) or a hydroxyl radical (HO^-^) [37]. Among them, H_2_O_2 _is one of the most stable ROS [38]. With both reducing and oxidizing properties, H_2_O_2 _has effects on almost all organisms, and can influence the life of every single cell. On one hand, H_2_O_2 _is highly reactive and toxic, and can lead to oxidative destruction of cells; on the other hand, it acts as a signaling molecule in regulating cell growth and development, cell proliferation, cell stress response, and signal transduction [39]. When accumulated at high enough concentrations, H_2_O_2 _can directly or indirectly oxidize enough of the cellular ascorbic acid and glutathione pool to alter the overall redox state of the cells. Such high concentrations of H_2_O_2 _can also damage a large variety of biomolecules such as lipids, proteins and nucleic acids that are essential for the activity and integrity of the cells [37,40]. As sessile organisms, plants have evolved a high degree of developmental plasticity to optimize their growth and reproduction in response to various biotic and abiotic stresses [41]. Under these conditions, the excessive H_2_O_2 _is efficiently scavenged by various antioxidative defense mechanisms in plant cells. The major ROS-scavenging enzymes include ascorbate peroxidase (APX), catalase (CAT), superoxide dismutase (SOD), glutathione peroxidase (GPX), monodehydroascorbate reductase (MDAR), Glutaredoxin (GLR) and peroxiredoxin (PrxR). Together with the antioxidants ascorbic acid and glutathione, these enzymes provide plant cells with highly efficient machinery for detoxifying H_2_O_2 _and other ROS [37,40,42].

In the present study, the expression levels of five genes involved in reactive oxygen species production and hemeostasis including superoxide dismutase (SOD), ascorbate peroxidase (APX), glutathione peroxidase (GPX), monodehydroascorbate reductase (MDAR) and peroxiredoxin (PrxR) were found to be higher in CSSL50-1 than those in Asominori (Figure 5B & Additional file 4 Table S2), suggesting that the antioxidative network in CSSL50-1 is activated. This result is consistent with the higher concentration of H_2_O_2 _in CSSL50-1. In the 15th day of grain-filling period, the concentration of H_2_O_2 _in CSSL50-1 reached 617 umol/g whereas that in Asominori is only 451.3 umol/g. The high concentration of H_2_O_2 _can provoke the defense system responsive to ROS (mainly H_2_O_2_) stress in CSSL50-1 to induce the expression of these antioxidative genes (including SOD, APX, GPX, MDAR and PrxR). Unexpectedly, another one antioxidative gene (GLR) was found to be down-regulated in CSSL50-1 compared to Asominori (Figure 5B & Additional file 4, Table S2), possibly due to more GSH to be needed for enhancing the functions of GST and Glx genes (Figure 5A).

In this study, we also found that the expression levels of GST, Glx and Trx genes are significantly higher in CSSL50-1compared to those in Asominori (Figure 5A & Additional file 4, Table S2). In contrast, LOX gene is down-regulated in CSSL50-1 (Figure 5B & Additional file 4, Table S2). GST is an antioxidative protein together with glutathione (GSH) to reduce oxidized biological macromolecule, and its expression can be strongly enhanced by abiotic and biotic stresses [25]. Glyoxalase I can convert toxic 2-oxoaldehydes into less reactive 2-hydroxyacids using GSH as a cofactor [43]. Thioredoxin (Trx) can reduce the oxidized proteins and peroxidative lipids [26]. However, lipoxygenase 5 (LOX5) is one member of a family of enzymes that deoxygenate unsaturated fatty acids, thus initiating lipoperoxidation of membranes [44]. These results suggest that the antioxidative level in CSSL50-1 is higher than that in Asominori.

### Grain chalkiness involves coordinated regulation of multiple pathways

It has been known for a long time that adverse environmental conditions can easily cause chalkiness in rice grain. High-temperatures, for example, have been shown to cause changes in the expression of genes involved in starch synthesis and directly correlated with the extent of gains chalkiness [5,45]. Drought stress, as well as sulphur deficiency which also activates antioxidation-related enzymes, can cause increased sucrose synthase activity and finally lead to the emergence of chalkiness too [33,46-49]. The reported studies show that exterior coercive conditions can break the oxidation-reduction balance, causing the change of carbohydrate metabolization in the rice plant, leading finally to the emergence of chalkiness. For example, 1) High-temperature stress can not only cause the change in the expression quantity of antioxidation-related genes, but also lead to the change in the expression quantity of carbon metabolism-related radical/protein, finally increasing the rice chalkiness [5,45] (Lin et al 2005; Yamakawa et al 2007). 2) Drought and coldness coercive conditions can also change the expression levels of carbon metabolization-related genes in the rice plant [50,51] (Gorantla et al 2007; Cheng et al 2007). Drought stress can also induce increase of sucrose synthase in the rice plant, finally leading to the emergence of chalkiness [33,46,47], which agrees with the result of this experiment; namely, the sucrose synthase activity and seed grain filling rate in high-chalkiness CSSL50-1 are remarkably higher than that in low-chalkiness Asominori. 3) For the rice plant with outside trauma treatment, salt stress, and ray irradiation, except the activation of cell-defense related genes, the expression quantity of carbon-metabolization related genes is also changed [52-54]. 4) Sulphur-deficiency can also bring about nutrition coercion of the rice plant, causing an increase in the activity of the rice's antioxidation-related enzymes, further leading to the emergence of rice chalkiness [48,49].

Reactive oxygen species (ROS), as represented by their most stable form H_2_O_2 _[38], play important roles as signaling molecules in regulating plant growth and development including cell proliferation, cell stress response, and signal transduction [40]. H_2_O_2 _is known to be involved in biotic and abiotic stress responses. The observed drastic increase in H_2_O_2 _levels in CSSL50-1 and the differential expression of several key regulatory genes involved on ROS production and scavenge collectively suggest that ROS may play a critical role in regulating rice endosperm chalkiness. Changes in H_2_O_2 _levels may affect multiple metabolism pathways in the rice endosperm, causing chalkiness phenotypic change. Further genetic and biochemical studies should further test such a possibility.

## Conclusion

Consistent with previous studies on the effect of adverse environmental conditions in causing chalky rice grain, our comparative transcriptome analysis of the caryopses of a near-isogenic line CSSL50-1 (with high chalkiness) and its low chalkiness parental line Asominori supports the notion that rice grain endosperm development is controlled by delicate, but complex genetic networks. Notably, several pathways related to signal transduction, cell rescue/defense, transcription, protein degradation, carbohydrate metabolism and redox homeostasis were found to be predominant among the differentially expressed genes, suggesting that formation of rice endosperm chalkiness may involve coordinated regulation of multiple pathways. Further refining of CSSL50-1 as a useful genetic material will help eventual cloning and engineering the major genes underlying the formation of rice grain chalkiness.

## Methods

### Plant material and growth

A japonica cultivar, Asominori, and its chromosome segment substitution line (CSSL50-1) were used in this study. CSSL50-1 is a near-isogenic line of Asominori with a substituted segment from the donor IR24 (an indica cultivar). Seventy-one F_7 _RILs were derived from a cross between Asominori and IR24 by single-seed descent [21]. To produce a series of CSSLs in a largely Asominori background, 19 selected RILs were crossed and then backcrossed with Asominori, without selection, until the BC_3_F_1 _generation. Sixty-six individuals were then selected at BC_3_F_1 _on the basis of a whole genome survey (116 RFLP loci) and were denoted as CSSL1-CSSL66 [55]. CSSL50 was observed to have high grain chalkiness characteristics. To further reduce the introgressed segment, CSSL50 was backcrossed with Asominori followed by two generations of self-pollination, and the progeny were evaluated using marker-assisted selection strategy. The homozygous line CSSL50-1 (BC_4_F_3_, Asominori/IR24//4*Asominori) was found to have high chalky grains and contains a small segment of IR24 chromosome 8 in a largely Asominori genetic background.

Four batches of seeds were sowed for both Asominori and CSSL50-1. The distance between the auricle of the flag-leaf (last leaf) and that of the penultimate leaf, or so-called Auricle distance (AD) was used as a non-destructive measurement to gauge rice flowering stage. CSSL50-1 started to flower when its AD reached 17 cm which was marked as day zero after flowering or DAF, whereas the AD for Asominori is 17.5 cm. Grain endosperms of the batches that flowered simultaneously for CSSL50-1 and Asominori were collected at 5, 10, 15, 20, 25, 30 and 35 DAF. The samples were immediately frozen in liquid nitrogen and stored at -80°C. RNA samples from 15 DAF endosperms were used for the DNA microarray analysis.

### Phenotype and physico-chemical properties of CSSL50-1 and Asominori grains

Seed starch granules was imaged as described in Kang et al. (2005) [4]. Samples for scanning electron microscopy (SEM) were pre-fixed with 3% glutaraldehyde for 3 h at room temperature, rinsed three times (15 min each) with 0.1 M sodium phosphate buffer (pH 6.8), and fixed overnight with 2% OsO_4 _at 4°C. The fixed samples were then washed three times (15 min each) with 0.1 M sodium phosphate buffer, dehydrated through an ethanol series, and incubated in a 1:3 (v:v) ethanol-isoamyl acetate mixture for 1 h. These samples were dried to a critical point, mounted on SEM stubs, and coated with gold. The mounted specimens were observed under SEM with an accelerating voltage of 10-20 kV. Fine structure of amylopectin was determined according to Fujita et al. (2006) [56].

### Determination of starch, amylase and sucrose content, chalkiness and RVA profiles

Starch content, amylose content and sucrose content of each accession were determined as Fujita et al. (2007) [57]. Percentage of grains with chalkiness (PGWC), area of chalky endosperm (ACE) and degree of endosperm chalkiness (DEC) were measured according to the method of Wan et al. (2005) [19]. To separate chalky from vitreous grains, 100 grains per entry were assessed on a chalkiness visualizer to calculate PGWC. Twenty chalky grains were then selected at random, and the ratio of the area of chalkiness to the area of the whole endosperm for each grain was evaluated by visual assessment on the chalkiness visualizer. The values were averaged and used as values for ACE. DEC was calculated as the product of PGWC × ACE. Rapid viscosity analyzer (RVA) profiles were characterized by six parameters such as peak paste viscosity (PKV), hot paste viscosity (HPV), cool paste viscosity (CPV), breakdown viscosity (BDV = PKV-HPV), consistency viscosity (CSV = CPV-HPV), and setback viscosity (SBV = CPV-PKV) as described in Brabender (1998) [58].

### Determination of photosynthesis efficiency

Maximum quantum efficiency of PS II photochemistry (Fv/Fm) and noncyclic electron flow (ΦPSII) of rice leaves were measured using a PAM-2000 portable PAM fluorometer (Walz Effeltrich, Germany) with the software DA-2000 (Heinz Walz). For each sample, at least nine leaves were measured.

### Measurement of sucrose synthase, AGPase, BE, and DBE

All enzymatic activity measurements were carried out in a 4°C cold chamber. In general, five immature rice grains without hull, pericarp, and embryo at the late-milking stage were homogenized in 1 mL of solution composed of 50 mM HEPES-NaOH (pH 7.4), 2 mM MgCl_2_, 50 mM 2-mercaptoethanol, and 12.5% (v/v) glycerol. The homogenate was centrifuged twice at 15, 000 g for 15 min. The supernatant was used as the crude enzyme extract [59,60]. The activities of sucrose synthase, AGPase, and BE were assayed as described [8,61]. Activity of DBE was measured using the methods of Nelson (1944) [62] and Somogyi (1952) [63].

### Measurement of grain H_2_O_2 _levels

H_2_O_2 _concentrations in Asominori and CSSL50-1 endosperm were measured according to Wan and Liu (2008) [39] with minor modifications. Briefly, rice endosperm of 15 DAF (1.0 g) were ground with a mortar and pestle in liquid nitrogen to fine powders and added to a 10-ml cuvette containing 8 ml of double distilled H_2_O and 2 ml of 25 mM titanium sulfate and then incubated for 1 h at room temperature. Oxidation of titanium sulfate was recorded by reading A410. Readings were converted to corresponding concentrations using a standard calibration plot.

### RNA extraction, GeneChip hybridization, and initial data analysis

RNA samples were processed according to Affymetrix manual. Total RNA was isolated using TRIzol reagent. RNA was then purified using an RNeasy spin column (Qiagen) and an on-column DNase treatment. Hybridization of Affrymetrix rice GeneChips and initial data collection were conducted at CapitalBio Corporation (Beijing, China). A total of 6 chips, with three biological replicates for each sample, were used in the assay. The hybridization data were analyzed using GeneChip Operating software (GCOS 1.4). A global scaling procedure was performed to normalize different arrays using dChip software, which incorporates a statistical model for expression array data at the probe level. The expression values were log_2 _transformed after calculating the expression index. Two-class unpaired method in the SAM (Significant Analysis of Microarrays) software (Tusher et al. 2001) [64] was used to identify the differentially expressed genes. One-way ANOVA was applied as an alternative statistic tool to further filter the differentially expressed genes.

The differentially expressed genes were classified using the Gene Ontology scheme http://www.geneontology.org/ and enriched GO terms were evaluated using Fisher's exact test. Rice annotation information was downloaded from AFFYMETRIX netaffx-annotation http://www.affymetrix.com/support/support_result.affx. Manual functional classification of differentially expressed genes was performed with the aid of the bioinformatics tool JAFA http://jafa.burnham.org. The microarray data has been deposited in the EMBL ArrayExpress database under the accession number E-MTAB-397.

### Semi-quantitative RT-PCR analysis

Five micrograms of RNA (15 DAF endosperms) were used for reverse-transcription (SuperScript II; Invitrogen). An aliquot of the first-strand cDNA mixture corresponding to 6.25 ng of total RNA was used as a template with 0.5 units of Taq polymerase (ExTaq; TaKaRa) in 50 uL volume. In general, after initial 5 min at 94°C, 30 cycles of 94°C for 30 s, 55°C for 30 s, 72°C for 1 min were performed with a final extension at 72°C for 10 min. Sequences of primers are listed in Additional file 5 (Table S3). PCR products were separated by electrophoresis in 1.5% agarose gels, stained with ethidium bromide, and visualized using the BioDoc-It system (UVP).

## Authors' contributions

XL, NS, HZ, and JW conceived and designed the experiments; XL and MZ analyzed the microarray data; XL TG, BM, XW performed part of the experiments; XL, HW, AL, LM, and JM prepared the manuscript. All authors have read and approved the final manuscript.

## Supplementary Material

Additional file 1 **Photosynthesis rates of rice leaves at various grain-filling stages of CSSL50-1 and Asominori**. (A) Maximum quantum efficiency of PS II photochemistry (Fv/Fm); (B) Noncyclic electron flow (ΦPSII). Click here for file

Additional file 2 **Scatter plot of signal intensities for all expressed probes on the Affymetrix microarray**. Normalized intensities correlation between expressed probes from Asominori (*x *axis) and CSSL50-1 (*y *axis). Click here for file

Additional file 3 **Enriched GO terms of 193 differentially expressed transcripts in CSSL50-1 and Asominori**. Enriched GO terms of 193 differentially expressed transcripts detected by Significant Analysis Microarray software (fold change > = 2, q value < = 5%). Click here for file

Additional file 4 **Functional classification of 623 differentially expressed genes between CSSL50-1 and Asominori**. Functional classification of 623 differentially expressed genes between CSSL50-1 and Asominori as detected by one-way ANOVA (P value <0.01). Click here for file

Additional file 5 **Sequence of primers used for RT-PCR**. A list of primers used for RT-PCR and the sequence information. Click here for file
