# Supplementary figures and images for: Transcriptome analysis of grain-filling caryopses reveals involvement of multiple regulatory pathways in chalky grain formation in rice
Source: BMC Genomics. 2010 Dec 30;11:730. doi: 10.1186/1471-2164-11-730 (PMC3023816; doi:10.1186/1471-2164-11-730)

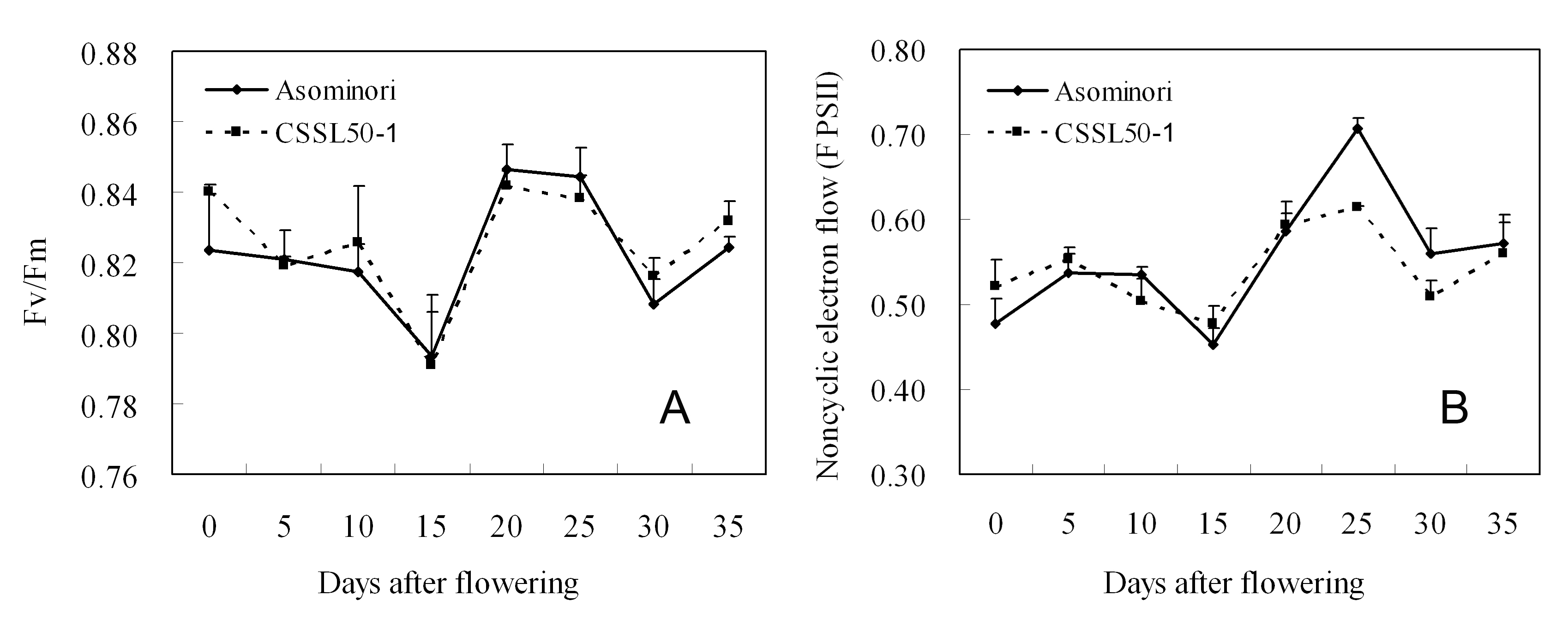

Supplement: Additional file 1 — Photosynthesis rates of rice leaves at various grain-filling stages of CSSL50-1 and Asominori. (A) Maximum quantum efficiency of PS II photochemistry (Fv/Fm); (B) Noncyclic electron flow (ΦPSII). [file 1471-2164-11-730-S1.JPEG]

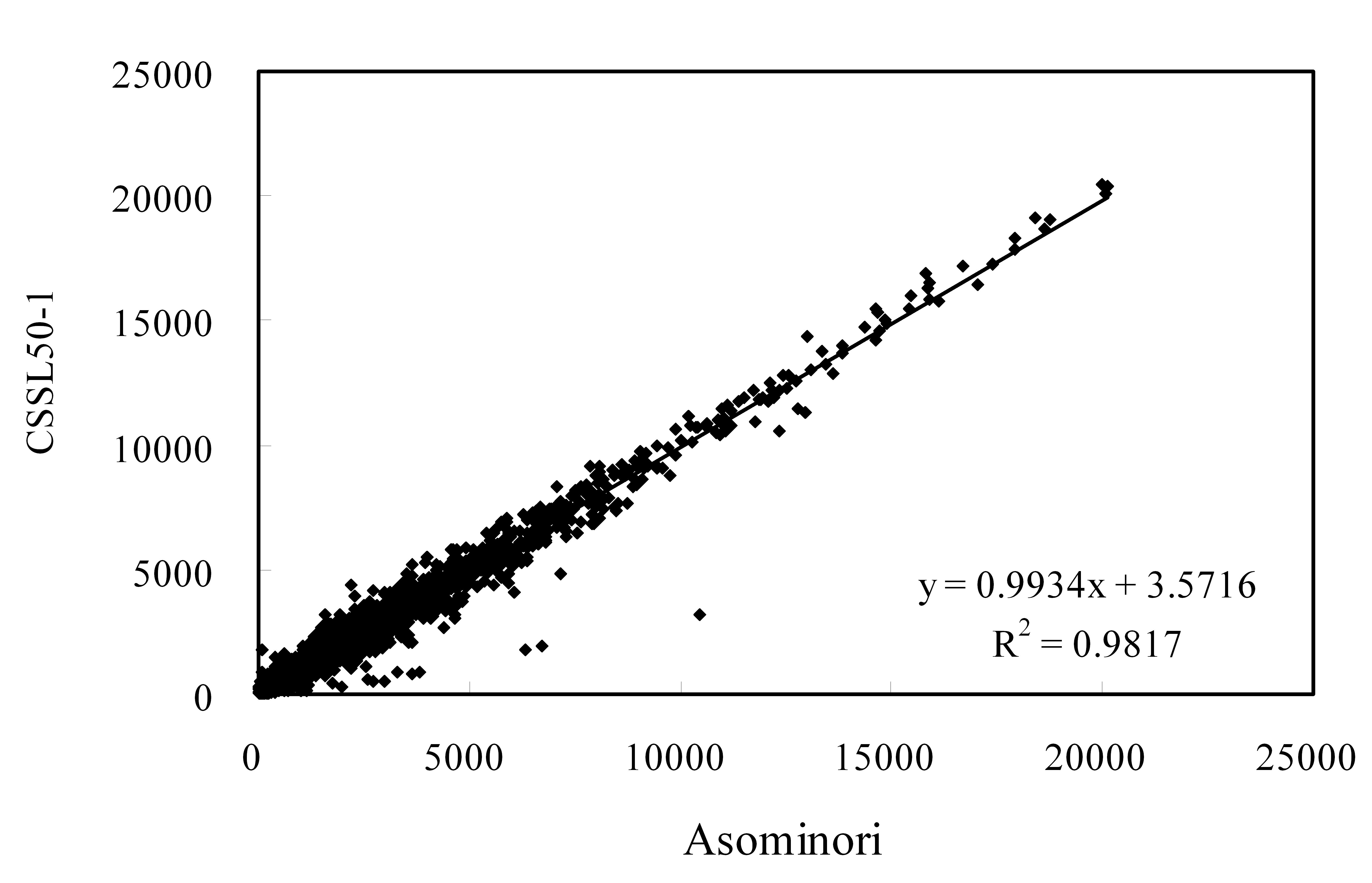

Supplement: Additional file 2 — Scatter plot of signal intensities for all expressed probes on the Affymetrix microarray. Normalized intensities correlation between expressed probes from Asominori (x axis) and CSSL50-1 (y axis). [file 1471-2164-11-730-S2.JPEG]
