# Supplementary material for: Transcriptome analysis of grain-filling caryopses reveals involvement of multiple regulatory pathways in chalky grain formation in rice
Source: BMC Genomics. 2010 Dec 30;11:730. doi: 10.1186/1471-2164-11-730 (PMC3023816; doi:10.1186/1471-2164-11-730)
Supplement: Additional file 3 — Enriched GO terms of 193 differentially expressed transcripts in CSSL50-1 and Asominori. Enriched GO terms of 193 differentially expressed transcripts detected by Significant Analysis Microarray software (fold change > = 2, q value < = 5%). [file 1471-2164-11-730-S3.DOC]

**Additional file 3**

**Table S1:** Enriched GO terms of 193 differentially expressed transcripts in CSSL50-1 and Asominori.^1^

| **Affy ID** | **Annotation** | **GB accession** | **fold** | **q-value (%)** | **GO accession** | **depth** |
| --- | --- | --- | --- | --- | --- | --- |
| **Biological_process** |  |  |  |  |  |  |
|  |  |  |  |  |  |  |
| ***lipid transport*** |  |  |  |  |  |  |
| Os.11169.1.S1_a_at | Nonspecific lipid-transfer protein | AK062667.1 | 3.76 | 3.17 | GO:0006869 | 3 |
| Os.28462.1.S1_s_at | Nonspecific lipid-transfer protein 5 precursor | AF051369.1 | 2.87 | 1.04 | GO:0006869 | 3 |
| Os.4223.1.S1_s_at | Transposon protein | AK103637.1 | 5.38 | 1.84 | GO:0006869 | 3 |
| Os.7771.1.S1_at | Protease inhibitor | AK103715.1 | 2.28 | 1.84 | GO:0006869 | 3 |
| Os.8827.1.S1_at | Nonspecific lipid-transfer protein precursor | AK071598.1 | 4.56 | 3.71 | GO:0006869 | 3 |
| Os.9672.1.S1_at |  | AU085959 | 0.45 | 1.80 | GO:0006869 | 3 |
| OsAffx.13437.1.S1_at |  | 9631.m04910 | 0.50 | 0.55 | GO:0006869 | 3 |
| ***lipid localization*** |  |  |  |  |  |  |
| Os.11169.1.S1_a_at | Nonspecific lipid-transfer protein | AK062667.1 | 3.76 | 3.17 | GO:0010876 | 3 |
| Os.28462.1.S1_s_at | Nonspecific lipid-transfer protein 5 precursor | AF051369.1 | 2.87 | 1.04 | GO:0010876 | 3 |
| Os.4223.1.S1_s_at | Transposon protein | AK103637.1 | 5.38 | 1.84 | GO:0010876 | 3 |
| Os.7771.1.S1_at | Protease inhibitor | AK103715.1 | 2.28 | 1.84 | GO:0010876 | 3 |
| Os.8827.1.S1_at | Nonspecific lipid-transfer protein precursor | AK071598.1 | 4.56 | 3.71 | GO:0010876 | 3 |
| Os.9672.1.S1_at |  | AU085959 | 0.45 | 1.80 | GO:0010876 | 3 |
| OsAffx.13437.1.S1_at |  | 9631.m04910 | 0.50 | 0.55 | GO:0010876 | 3 |
| ***response to stress*** | |  |  |  |  |  |
| Os.8165.1.S1_at | Wound/stress protein | AK065736.1 | 3.12 | 0.69 | GO:0006950 | 2 |
| Os.47418.1.S1_a_at | Peroxidase 64 precursor | AK099584.1 | 4.15 | 1.04 | GO:0006950 | 2 |
| Os.4987.1.S1_at | L-ascorbate peroxidase 3 | AY382617.1 | 0.25 | 3.71 | GO:0006950 | 2 |
| Os.37616.1.S1_at | HSP90-like protein mRNA | AY077617.1 | 2.79 | 1.84 | GO:0006950 | 2 |
| Os.51718.1.S1_at | Dehydrin RAB 16B | AK063517.1 | 3.88 | 3.17 | GO:0006950 | 2 |
| OsAffx.29642.1.S1_at | AP2 domain containing protein | 9636.m04399 | 0.49 | 0.34 | GO:0006950 | 2 |
| Os.22730.1.S1_at | 17.4 kDa class I heat shock protein | AK069547.1 | 2.05 | 1.84 | GO:0006950 | 2 |
| Os.37773.1.S1_at | 17.4 kDa class I heat shock protein | AU165294 | 2.32 | 1.58 | GO:0006950 | 2 |
| Os.4775.1.S1_at | 16.9 kDa class I heat shock protein | AK121025.1 | 4.51 | 2.18 | GO:0006950 | 2 |
| Os.10651.1.S1_at |  | AK121915.1 | 2.68 | 3.17 | GO:0006950 | 2 |
| Os.16023.1.S1_at |  | AK107251.1 | 0.45 | 0.34 | GO:0006950 | 2 |
| Os.8195.1.S1_at |  | AU165131 | 2.11 | 1.84 | GO:0006950 | 2 |
| ***carbohydrate metabolic process*** | |  |  |  |  |  |
| Os.53946.1.S1_at | NAD dependent epimerase/dehydratase family protein | AK100965.1 | 0.43 | 0.34 | GO:0005975 | 3 |
| Os.54304.1.S1_at | Lectin precursor | M24504.1 | 5.53 | 3.71 | GO:0005975 | 3 |
| Os.11714.1.S1_at | Glycosyl hydrolases family 17 protein | AK102185.1 | 2.10 | 0.69 | GO:0005975 | 3 |
| Os.7566.1.S1_at | Glycosyl hydrolases family 16 protein | AK059277.1 | 2.58 | 1.58 | GO:0005975 | 3 |
| Os.15799.1.S1_at | Glycosyl hydrolase family 1 protein | AY129294.1 | 2.02 | 3.71 | GO:0005975 | 3 |
| Os.23365.1.S1_at | Glucose-6-phosphate isomerase | AK107494.1 | 3.21 | 0.00 | GO:0005975 | 3 |
| Os.12593.1.S1_s_at | Alpha-amylase isozyme 3E precursor | AK064300.1 | 7.38 | 1.84 | GO:0005975 | 3 |
| Os.25572.1.S1_at | Aldose 1-epimerase family protein | AK109600.1 | 5.18 | 1.04 | GO:0005975 | 3 |
| Os.17195.1.A1_at |  | AK066850.1 | 0.50 | 2.18 | GO:0005975 | 3 |
| Os.24051.1.S1_x_at |  | AK110902.1 | 0.44 | 1.80 | GO:0005975 | 3 |
| Os.35154.1.S1_x_at |  | AK068772.1 | 0.43 | 0.00 | GO:0005975 | 3 |
| ***macromolecule localization*** | |  |  |  |  |  |
| Os.11169.1.S1_a_at | Nonspecific lipid-transfer protein | AK062667.1 | 3.76 | 3.17 | GO:0033036 | 2 |
| Os.28462.1.S1_s_at | Nonspecific lipid-transfer protein 5 precursor | AF051369.1 | 2.87 | 1.04 | GO:0033036 | 2 |
| Os.4223.1.S1_s_at | Transposon protein | AK103637.1 | 5.38 | 1.84 | GO:0033036 | 2 |
| Os.7771.1.S1_at | Protease inhibitor | AK103715.1 | 2.28 | 1.84 | GO:0033036 | 2 |
| Os.8827.1.S1_at | Nonspecific lipid-transfer protein precursor | AK071598.1 | 4.56 | 3.71 | GO:0033036 | 2 |
| Os.9672.1.S1_at |  | AU085959 | 0.45 | 1.80 | GO:0033036 | 2 |
| OsAffx.13437.1.S1_at |  | 9631.m04910 | 0.50 | 0.55 | GO:0033036 | 2 |
| ***regulation of transcription, DNA-dependent*** | |  |  |  |  |  |
| Os.12994.1.S1_at | Myb-like DNA-binding domain containing protein | AB064519.1 | 2.12 | 0.69 | GO:0006355 | 5 |
| Os.24952.1.S1_at | Two-component response regulator ARR3 | AK059734.1 | 0.38 | 0.00 | GO:0006355 | 5 |
| Os.29967.1.S1_at | GAF domain containing protein | AY434734.1 | 3.75 | 1.58 | GO:0006355 | 5 |
| Os.40106.1.A1_s_at | Retinoblastoma | CF328544 | 2.07 | 1.04 | GO:0006355 | 5 |
| Os.4893.1.S1_at | AP2 domain containing protein | AK100184.1 | 2.24 | 2.18 | GO:0006355 | 5 |
| Os.51648.1.S1_at | Homeobox domain containing protein | AK063262.1 | 2.33 | 3.17 | GO:0006355 | 5 |
| Os.55050.1.S1_at | Response regulator receiver domain containing protein | AK107384.1 | 0.27 | 0.00 | GO:0006355 | 5 |
| OsAffx.29642.1.S1_at | AP2 domain containing protein | 9636.m04399 | 0.49 | 0.34 | GO:0006355 | 5 |
| ***regulation of RNA metabolic process*** | |  |  |  |  |  |
| Os.12994.1.S1_at | Myb-like DNA-binding domain containing protein | AB064519.1 | 2.12 | 0.69 | GO:0051252 | 4 |
| Os.24952.1.S1_at | Two-component response regulator ARR3 | AK059734.1 | 0.38 | 0.00 | GO:0051252 | 4 |
| Os.29967.1.S1_at | GAF domain containing protein | AY434734.1 | 3.75 | 1.58 | GO:0051252 | 4 |
| Os.40106.1.A1_s_at | Retinoblastoma | CF328544 | 2.07 | 1.04 | GO:0051252 | 4 |
| Os.4893.1.S1_at | AP2 domain containing protein | AK100184.1 | 2.24 | 2.18 | GO:0051252 | 4 |
| Os.51648.1.S1_at | Homeobox domain containing protein | AK063262.1 | 2.33 | 3.17 | GO:0051252 | 4 |
| Os.55050.1.S1_at | Response regulator receiver domain containing protein | AK107384.1 | 0.27 | 0.00 | GO:0051252 | 4 |
| OsAffx.29642.1.S1_at | AP2 domain containing protein | 9636.m04399 | 0.49 | 0.34 | GO:0051252 | 4 |
| ***transcription, DNA-dependent*** | |  |  |  |  |  |
| Os.12994.1.S1_at | Myb-like DNA-binding domain containing protein | AB064519.1 | 2.12 | 0.69 | GO:0006351 | 5 |
| Os.24952.1.S1_at | Two-component response regulator ARR3 | AK059734.1 | 0.38 | 0.00 | GO:0006351 | 5 |
| Os.29967.1.S1_at | GAF domain containing protein | AY434734.1 | 3.75 | 1.58 | GO:0006351 | 5 |
| Os.40106.1.A1_s_at | Retinoblastoma | CF328544 | 2.07 | 1.04 | GO:0006351 | 5 |
| Os.4893.1.S1_at | AP2 domain containing protein | AK100184.1 | 2.24 | 2.18 | GO:0006351 | 5 |
| Os.51648.1.S1_at | Homeobox domain containing protein | AK063262.1 | 2.33 | 3.17 | GO:0006351 | 5 |
| Os.55050.1.S1_at | Response regulator receiver domain containing protein | AK107384.1 | 0.27 | 0.00 | GO:0006351 | 5 |
| OsAffx.29642.1.S1_at | AP2 domain containing protein | 9636.m04399 | 0.49 | 0.34 | GO:0006351 | 5 |
| ***RNA biosynthetic process*** | |  |  |  |  |  |
| Os.12994.1.S1_at | Myb-like DNA-binding domain containing protein | AB064519.1 | 2.12 | 0.69 | GO:0032774 | 4 |
| Os.24952.1.S1_at | Two-component response regulator ARR3 | AK059734.1 | 0.38 | 0.00 | GO:0032774 | 4 |
| Os.29967.1.S1_at | GAF domain containing protein | AY434734.1 | 3.75 | 1.58 | GO:0032774 | 4 |
| Os.40106.1.A1_s_at | Retinoblastoma | CF328544 | 2.07 | 1.04 | GO:0032774 | 4 |
| Os.4893.1.S1_at | AP2 domain containing protein | AK100184.1 | 2.24 | 2.18 | GO:0032774 | 4 |
| Os.51648.1.S1_at | Homeobox domain containing protein | AK063262.1 | 2.33 | 3.17 | GO:0032774 | 4 |
| Os.55050.1.S1_at | Response regulator receiver domain containing protein | AK107384.1 | 0.27 | 0.00 | GO:0032774 | 4 |
| OsAffx.29642.1.S1_at | AP2 domain containing protein | 9636.m04399 | 0.49 | 0.34 | GO:0032774 | 4 |
| ***primary metabolic process*** | |  |  |  |  |  |
| Os.10194.1.S1_at | U-box domain containing protein | AK100423.1 | 0.20 | 0.00 | GO:0044238 | 2 |
| Os.54140.1.S1_at | Ubiquitin carboxyl-terminal hydrolase 1 | AK102160.1 | 0.14 | 0.00 | GO:0044238 | 2 |
| Os.24952.1.S1_at | Two-component response regulator ARR3 | AK059734.1 | 0.38 | 0.00 | GO:0044238 | 2 |
| Os.11412.1.S1_a_at | S-adenosylmethionine decarboxylase proenzyme | AK102445.1 | 2.34 | 1.58 | GO:0044238 | 2 |
| Os.50495.1.S1_at | Retrotransposon protein | AK121448.1 | 0.06 | 0.00 | GO:0044238 | 2 |
| Os.40106.1.A1_s_at | Retinoblastoma | CF328544 | 2.07 | 1.04 | GO:0044238 | 2 |
| Os.55050.1.S1_at | Response regulator receiver domain containing protein | AK107384.1 | 0.27 | 0.00 | GO:0044238 | 2 |
| Os.12634.1.S1_at | Protein kinase domain containing protein | AK111766.1 | 0.48 | 0.34 | GO:0044238 | 2 |
| Os.6085.1.S1_at | Protein kinase domain containing protein | AK105946.1 | 0.48 | 1.80 | GO:0044238 | 2 |
| Os.54867.1.S1_at | Protein kinase APK1B | AK106955.1 | 0.35 | 0.00 | GO:0044238 | 2 |
| Os.57422.1.S1_x_at | Oxysterol-binding protein | CA764876 | 0.46 | 1.04 | GO:0044238 | 2 |
| Os.53946.1.S1_at | NAD dependent epimerase/dehydratase family protein | AK100965.1 | 0.43 | 0.34 | GO:0044238 | 2 |
| Os.12994.1.S1_at | Myb-like DNA-binding domain containing protein | AB064519.1 | 2.12 | 0.69 | GO:0044238 | 2 |
| Os.8586.1.S1_at | Leucine-rich repeat transmembrane protein kinase | AK103914.1 | 2.26 | 1.04 | GO:0044238 | 2 |
| OsAffx.17422.1.S1_at | Leucine Rich Repeat family protein | 9636.m04000 | 2.03 | 1.84 | GO:0044238 | 2 |
| Os.54304.1.S1_at | Lectin precursor | M24504.1 | 5.53 | 3.71 | GO:0044238 | 2 |
| Os.37616.1.S1_at | HSP90-like protein mRNA | AY077617.1 | 2.79 | 1.84 | GO:0044238 | 2 |
| Os.51648.1.S1_at | Homeobox domain containing protein | AK063262.1 | 2.33 | 3.17 | GO:0044238 | 2 |
| Os.20204.2.S1_a_at | Histone deacetylase | AF513384.1 | 2.24 | 3.71 | GO:0044238 | 2 |
| Os.55174.1.S1_at | Helix-loop-helix DNA-binding domain containing protein | AK107626.1 | 5.02 | 0.69 | GO:0044238 | 2 |
| Os.11714.1.S1_at | Glycosyl hydrolases family 17 protein | AK102185.1 | 2.10 | 0.69 | GO:0044238 | 2 |
| Os.7566.1.S1_at | Glycosyl hydrolases family 16 protein | AK059277.1 | 2.58 | 1.58 | GO:0044238 | 2 |
| Os.15799.1.S1_at | Glycosyl hydrolase family 1 protein | AY129294.1 | 2.02 | 3.71 | GO:0044238 | 2 |
| Os.23365.1.S1_at | Glucose-6-phosphate isomerase | AK107494.1 | 3.21 | 0.00 | GO:0044238 | 2 |
| Os.18450.1.S1_s_at | GDSL-motif lipase/hydrolase family protein | AK100958.1 | 2.35 | 3.71 | GO:0044238 | 2 |
| Os.8707.3.S1_x_at | GDSL-motif lipase/hydrolase family protein | AK103825.1 | 2.42 | 3.71 | GO:0044238 | 2 |
| Os.29967.1.S1_at | GAF domain containing protein | AY434734.1 | 3.75 | 1.58 | GO:0044238 | 2 |
| Os.11516.1.S1_s_at | D-3-phosphoglycerate dehydrogenase | BI807619 | 2.79 | 0.00 | GO:0044238 | 2 |
| OsAffx.29514.1.S1_at | D-3-phosphoglycerate dehydrogenase | 9636.m03484 | 2.69 | 0.00 | GO:0044238 | 2 |
| Os.12642.1.S1_at | Calcium-dependent protein kinase | AK103306.1 | 0.34 | 0.34 | GO:0044238 | 2 |
| Os.4893.1.S1_at | AP2 domain containing protein | AK100184.1 | 2.24 | 2.18 | GO:0044238 | 2 |
| OsAffx.29642.1.S1_at | AP2 domain containing protein | 9636.m04399 | 0.49 | 0.34 | GO:0044238 | 2 |
| Os.12593.1.S1_s_at | Alpha-amylase isozyme 3E precursor | AK064300.1 | 7.38 | 1.84 | GO:0044238 | 2 |
| Os.25572.1.S1_at | Aldose 1-epimerase family protein | AK109600.1 | 5.18 | 1.04 | GO:0044238 | 2 |
| Os.12342.1.S1_at |  | AK072775.1 | 0.47 | 3.71 | GO:0044238 | 2 |
| Os.17195.1.A1_at |  | AK066850.1 | 0.50 | 2.18 | GO:0044238 | 2 |
| Os.23932.1.A1_at |  | AK108082.1 | 3.49 | 1.84 | GO:0044238 | 2 |
| Os.24051.1.S1_x_at |  | AK110902.1 | 0.44 | 1.80 | GO:0044238 | 2 |
| Os.35154.1.S1_x_at |  | AK068772.1 | 0.43 | 0.00 | GO:0044238 | 2 |
|  |  |  |  |  |  |  |
| **Molecular function** |  |  |  |  |  |  |
|  |  |  |  |  |  |  |
| ***hydrolase activity, hydrolyzing O-glycosyl compounds*** | |  |  |  |  |  |
| Os.54304.1.S1_at | Lectin precursor | M24504.1 | 5.53 | 3.71 | GO:0004553 | 4 |
| Os.11714.1.S1_at | Glycosyl hydrolases family 17 protein | AK102185.1 | 2.10 | 0.69 | GO:0004553 | 4 |
| Os.15799.1.S1_at | Glycosyl hydrolase family 1 protein | AY129294.1 | 2.02 | 3.71 | GO:0004553 | 4 |
| Os.12593.1.S1_s_at | Alpha-amylase isozyme 3E precursor | AK064300.1 | 7.38 | 1.84 | GO:0004553 | 4 |
| Os.17195.1.A1_at |  | AK066850.1 | 0.50 | 2.18 | GO:0004553 | 4 |
| Os.24051.1.S1_x_at |  | AK110902.1 | 0.44 | 1.80 | GO:0004553 | 4 |
| Os.35154.1.S1_x_at |  | AK068772.1 | 0.43 | 0.00 | GO:0004553 | 4 |
| ***oxidoreductase activity*** | |  |  |  |  |  |
| Os.37946.1.S1_at | Ribulose bisphosphate carboxylase | AK059909.1 | 4.12 | 1.84 | GO:0016491 | 2 |
| Os.47418.1.S1_a_at | Peroxidase 64 precursor | AK099584.1 | 4.15 | 1.04 | GO:0016491 | 2 |
| Os.9749.1.S1_at | Monodehydroascorbate reductase | AK102459.1 | 2.48 | 0.00 | GO:0016491 | 2 |
| Os.4987.1.S1_at | L-ascorbate peroxidase 3 | AY382617.1 | 0.25 | 3.71 | GO:0016491 | 2 |
| Os.12979.3.S1_x_at | IQ calmodulin-binding motif family protein | NM_190466.1 | 0.46 | 1.80 | GO:0016491 | 2 |
| Os.14372.1.S1_at | Glutathione S-transferase | AK102889.1 | 3.10 | 2.18 | GO:0016491 | 2 |
| Os.10126.1.S1_at | EF hand family protein | BI806177 | 0.44 | 3.17 | GO:0016491 | 2 |
| Os.8531.1.S1_at | DSBA-like thioredoxin domain containing protein | AK059943.1 | 4.54 | 3.71 | GO:0016491 | 2 |
| Os.11516.1.S1_s_at | D-3-phosphoglycerate dehydrogenase | BI807619 | 2.79 | 0.00 | GO:0016491 | 2 |
| OsAffx.29514.1.S1_at | D-3-phosphoglycerate dehydrogenase | 9636.m03484 | 2.69 | 0.00 | GO:0016491 | 2 |
| Os.53717.1.S1_at | Cytochrome P450 family protein | AK099468.1 | 2.33 | 3.17 | GO:0016491 | 2 |
| Os.12201.1.S1_at | 1-aminocyclopropane-1-carboxylate oxidase 1 | AK065039.1 | 4.34 | 1.84 | GO:0016491 | 2 |
| Os.16162.1.S1_at |  | AK070561.1 | 2.61 | 0.00 | GO:0016491 | 2 |
| Os.50501.1.S1_at |  | AK121472.1 | 0.46 | 0.55 | GO:0016491 | 2 |
| Os.53601.1.S1_at |  | AK073522.1 | 0.16 | 0.00 | GO:0016491 | 2 |
| Os.55404.1.S1_at |  | AK108019.1 | 0.48 | 2.18 | GO:0016491 | 2 |
| Os.55408.1.S1_at |  | AK108024.1 | 7.40 | 1.58 | GO:0016491 | 2 |
| Os.57411.1.S1_at |  | AK111482.1 | 0.39 | 0.00 | GO:0016491 | 2 |
| OsAffx.26329.1.S1_at |  | 9632.m03328 | 0.22 | 0.00 | GO:0016491 | 2 |
| OsAffx.4769.1.S1_at |  | 9634.m00792 | 2.05 | 0.69 | GO:0016491 | 2 |
|  | | | | | | |

^1^Enriched GO terms of 193 differentially expressed transcripts detected by Significant Analysis Microarray software (fold change >=2, q value <= 5%).
