# Supplementary material for: Transcriptome analysis of grain-filling caryopses reveals involvement of multiple regulatory pathways in chalky grain formation in rice
Source: BMC Genomics. 2010 Dec 30;11:730. doi: 10.1186/1471-2164-11-730 (PMC3023816; doi:10.1186/1471-2164-11-730)
Supplement: Additional file 4 — Functional classification of 623 differentially expressed genes between CSSL50-1 and Asominori. Functional classification of 623 differentially expressed genes between CSSL50-1 and Asominori as detected by one-way ANOVA (P value <0.01). [file 1471-2164-11-730-S4.DOC]

**Additional file 4**

**Table S2: Functional classification of 623 differentially expressed genes between CSSL50-1 and Asominori. ^1^**

| NCBI No. | Transcript Assignments | *P* values | Ratio | Average signaling values | |
| --- | --- | --- | --- | --- | --- |
| ***Up-regulated genes*** | |  |  |  |  |
| **Amide acid metabolism** | |  |  | CSSL50 | Asominori |
| AK099473.1 | ACT domain-containing protein | 0.0005 | 1.219 | 1522.8±10.9 | 1249.2±44.2 |
| AK068397.1 | ACT domain-containing protein | 0.0047 | 1.436 | 113.6±6.8 | 79.1±8.0 |
| AK060835.1 | Alanine aminotransferase 2 | 0.0049 | 1.269 | 325.3±21.1 | 256.4±2.2 |
| AK107237.1 | Alanine aminotransferase 2 | 0.0059 | 1.880 | 691.4±98.9 | 367.9±34.6 |
| AK074023.1 | Amino acid transporter family protein | 0.0033 | 1.461 | 81.9±3.7 | 56.1±6.1 |
| AK099234.1 | Aminomethyltransferase,mitochondrial precursor | 0.0018 | 1.313 | 365.8±19.4 | 278.6±6.3 |
| AK100259.1 | Aminotransferase, class III family protein | 0.0037 | 1.412 | 100.1±6.3 | 70.9±5.5 |
| AK107879.1 | Anthranilate synthase component II | 0.0002 | 1.879 | 1001.4±50.4 | 532.9±37.8 |
| AK101488.1 | Arginase | 0.0004 | 1.154 | 1563.2±20.4 | 1354.2±26.4 |
| NM_184454.1 | Arginyl-tRNA synthetase family protein | 0.004 | 1.317 | 56.2±3.1 | 42.7±2.4 |
| D83378.1 | Asparagine synthetase, putative | 0.0031 | 1.154 | 3603.9±74.8 | 3122.6±107.3 |
| AF073696.1 | Cysteine synthase | 0.0025 | 1.614 | 110.2±10.3 | 68.3±2.8 |
| AK065652.1 | Cysteine synthase,chloroplast precursor | 0.0025 | 1.475 | 463.4±29.1 | 314.1±24.7 |
| AK099152.1 | Nitrogen regulatory protein P-II | 0.0074 | 1.161 | 506.8±15.5 | 436.4±18.7 |
| AK071754.1 | Phytochelatin synthase | 0.0099 | 1.339 | 275.6±25.5 | 205.9±5.8 |
| AK105587.1 | Saccharopine dehydrogenase family protein | 0.0014 | 1.133 | 1050.5±27.1 | 926.8±3.2 |
| AF067194.1 | S-adenosylmethionine decarboxylase proenzyme | 0.003 | 1.443 | 2979.7±241.2 | 2064.5±51.4 |
| AK102681.1 | WGR domain containing protein | 0.0083 | 1.842 | 1382.4±205.4 | 750.6±91.7 |
| **Carbohydrate metabolism** | |  |  |  |  |
| AF367205.1 | 1-deoxy-D-xylulose 5-phosphate reductoisomerase,chloroplast precursor | 0.0056 | 1.233 | 297.8±9.2 | 241.6±15.4 |
| AK063214.1 | Acetyltransferase,GNAT family protein | 0.0034 | 1.145 | 1180.2±20.0 | 1031.1±36.3 |
| AK063966.1 | Alpha-D-xylosidase | 0.0088 | 1.828 | 82.2±5.1 | 45.0±12.5 |
| AK102526.1 | Alpha-L-arabinofuranosidase C-terminus family protein | 0.0030 | 1.482 | 173.0±8.1 | 116.7±12.8 |
| AK099443.1 | Chalcone-flavanone isomerase family protein | 0.0083 | 1.815 | 78.1±12.3 | 43.0±2.0 |
| BI807619 | D-3-phosphoglycerate dehydrogenase | 0.0011 | 2.789 | 248.1±31.2 | 89.0±9.5] |
| 9636.m03484 | D-3-phosphoglycerate dehydrogenase, chloroplast precursor | 0.0013 | 2.687 | 158.5±19.3 | 59.0±9.0 |
| AK121222.1 | Dihydroneopterin aldolase | 0.0005 | 1.586 | 754.2±25.2 | 475.6±39.6 |
| AK099387.1 | Fructose-bisphosphate aldolase 1,chloroplast precursor | 0.0058 | 1.365 | 1528.0±91.7 | 1119.2±94.5 |
| AK107494.1 | Glucose-6-phosphate isomerase | 0.0001 | 3.211 | 442.4±17.4 | 137.8±3.6 |
| AK121407.1 | Glycoside hydrolase family 28 protein | 0.0042 | 1.144 | 147.9±3.8 | 129.3±4.0 |
| AK066300.1 | Glycosyl hydrolase family 3 N terminal domain containing protein | 0.0042 | 1.409 | 177.9±1.6 | 126.2±15.1 |
| CR282303 | starch debranching enzyme | 0.0100 | 1.161 | 7546.1±346.4 | 6497.5±288.2 |
| AK122106.1 | Glycosyltransferase 5 | 0.0036 | 1.399 | 77.0±3.8 | 55.0±4.9 |
| AK106478.1 | Mitochondrial glycoprotein | 0.0042 | 1.208 | 95.7±1.3 | 79.2±4.7 |
| AK070705.1 | Phosphoglycerate kinase,cytosolic | 0.0091 | 1.167 | 6001.5±146.8 | 5142.5±278.6 |
| AK071782.1 | Pyruvate dehydrogenase E1 component beta subunit, mitochondrial precursor | 0.0013 | 1.576 | 436.0±17.5 | 276.7±29.2 |
| AK071525.1 | Sucrose-phosphatase | 0.0047 | 1.310 | 286.4±11.3 | 218.6±17.3 |
| AK121341.1 | Sucrose-phosphate synthase | 0.0100 | 1.595 | 151.0±17.5 | 94.7±12.5 |
| **Cell cycle/biogenesis** | |  |  |  |  |
| AK121803.1 | 65kD microtubule associated protein | 0.0021 | 1.635 | 303.5±22.3 | 185.7±18.4 |
| AK072559.1 | Annexin-like protein RJ4 | 0.0082 | 1.147 | 1122.8±48.9 | 978.9±15.2 |
| AK066942.1 | Circadian clock coupling factor | 0.0055 | 1.137 | 333.2±9.9 | 293.0±8.1 |
| AK103765.1 | Cyclin, C-terminal domain containing protein | 0.0051 | 1.168 | 117.3±2.2 | 100.4±4.8 |
| AK070907.1 | ENT domain containing protein | 0.0067 | 1.126 | 213.7±1.9 | 189.8±7.8 |
| AK099313.1 | Fasciclin domain containing protein | 0.0067 | 1.301 | 381.1±26.2 | 293.0±13.7 |
| AK103508.1 | Growth-regulating factor | 0.0075 | 1.219 | 111.0±6.3 | 91.0±2.9 |
| AK066667.1 | Histone deacetylase 1A | 0.001 | 1.593 | 899.4±65.6 | 564.5±15.6 |
| U74296.1 | Linker histone H1 and H5 family protein | 0.0036 | 1.338 | 4661.2±300.7 | 3483.0±142.9 |
| AB079873.1 | Meiotic recombination protein DMC1 homolog | 0.0034 | 1.355 | 138.2±8.4 | 102.0±5.6 |
| AK071369.1 | MSP domain containing protein | 0.008 | 1.264 | 332.4±21.2 | 263.0±12.2 |
| AF068333.1 | Phytosulfokines 5 precursor | 0.0066 | 1.698 | 107.7±12.0 | 63.5±8.6 |
| AK070122.1 | Seh1 | 0.0077 | 1.312 | 385.4±18.8 | 293.7±26.0 |
| **Cell rescue/defense** | |  |  |  |  |
| AK061054.1 | Allinase, C-terminal domain containing protein | 0.0075 | 1.513 | 76.5±8.9 | 50.6±1.5 |
| AK103582.1 | Betaine-aldehyde dehydrogenase | 0.0037 | 1.476 | 1362.5±97.2 | 923.2±78.7 |
| AK100163.1 | Cytochrome P450 family protein | 0.0042 | 1.297 | 2855.9±79.0 | 2201.2±175.9 |
| D26538.1 | Dehydrin family protein | 0.0026 | 1.745 | 498.0±55.1 | 285.4±3.3 |
| AK102608.1 | DNA repair protein Rad4 containing protein | 0.0054 | 1.266 | 117.9±6.6 | 93.2±4.3 |
| AK102565.1 | DnaJ domain containing protein | 0.0058 | 1.303 | 1380.3±94.6 | 1059.0±41.8 |
| AK101574.1 | DnaJ domain containing protein | 0.0011 | 1.716 | 417.7±34.7 | 243.4±8.0 |
| AK063149.1 | Harpin-induced protein 1 containing protein | 0.0006 | 1.221 | 114.4±2.9 | 93.6±2.2 |
| AK101015.1 | HEAT repeat family protein | 0.0004 | 1.614 | 2784.3±157.5 | 1725.2±70.5 |
| AK073949.1 | Heavy metal-associated domain containing protein | 0.0033 | 1.412 | 62.0±4.9 | 43.9±0.9 |
| AK072765.1 | Heparanase-like protein 2 precursor | 0.001 | 2.024 | 405.5±37.5 | 200.3±16.4 |
| AK103871.1 | HhH-GPD superfamily base excision DNA repair protein | 0.003 | 1.656 | 93.2±6.4 | 56.3±7.6 |
| AK121005.1 | Leucine Rich Repeat family protein | 0.0092 | 1.294 | 295.1±22.7 | 228.1±9.4 |
| NM_184266.1 | Leucine Rich Repeat family protein | 0.0048 | 1.581 | 70.5±6.9 | 44.6±3.8 |
| AK119808.1 | Leucine-rich repeat transmembrane protein kinase | 0.0042 | 1.665 | 107.4±12.1 | 64.5±3.7 |
| AK121347.1 | MLO-like protein 1 | 0.0001 | 1.465 | 196.2±7.0 | 133.9±1.0 |
| AK065402.1 | Molybdenum cofactor biosynthesis protein 1 A | 0.0055 | 1.289 | 54.9±2.4 | 42.6±3.1 |
| C22605 | NB-ARC domain containing protein | 0.0077 | 1.337 | 63.0±4.9 | 47.2±2.6 |
| AF456246.1 | NB-ARC domain containing protein | 0.0015 | 1.370 | 60.0±3.3 | 43.8±1.5 |
| AK102351.1 | NB-ARC domain containing protein | 0.0065 | 1.399 | 68.8±6.5 | 49.1±0.6 |
| AK101608.1 | Nodulin | 0.0086 | 1.358 | 175.5±13.7 | 129.3±9.6 |
| AK068437.1 | Pectinacetylesterase family protein | 0.0084 | 1.482 | 51.9±1.7 | 35.0±5.8 |
| AK070039.1 | Pentatricopeptide | 0.0012 | 1.156 | 256.7±7.1 | 222.0±1.7 |
| AK068524.1 | Pentatricopeptide | 0.0082 | 1.253 | 118.8±8.0 | 94.8±3.0 |
| AK063792.1 | Pentatricopeptide | 0.0056 | 1.487 | 195.7±7.2 | 131.6±19.2 |
| AK120188.1 | PPR986-12 | 0.0088 | 1.281 | 140.3±10.8 | 109.5±2.8 |
| AK102604.1 | Remorin, C-terminal region family protein | 0.0051 | 1.108 | 151.1±4.2 | 136.3±1.7 |
| CF328544 | Retinoblastoma | 0.0051 | 2.067 | 52.1±6.7 | 25.2±5.0 |
| AK070273.1 | Senescence-associated protein | 0.0046 | 1.491 | 240.3±23.5 | 161.2±4.3 |
| AK062315.1 | SNARE domain containing protein | 0.0019 | 1.591 | 112.1±7.4 | 70.5±6.6 |
| AK068329.1 | SOUL | 0.0054 | 1.425 | 515.8±22.4 | 362.1±43.2 |
| AK102887.1 | SOUL heme-binding family protein | 0.0077 | 1.424 | 767.0±36.7 | 538.6±70.9 |
| AK062552.1 | Steroid binding protein 3 | 0.0003 | 1.152 | 2372.7±44.2 | 2059.8±12.1 |
| AK065178.1 | Wound/stress protein | 0.0062 | 1.476 | 1157.7±110.9 | 784.2±52.7 |
| **Energy pathway** | |  |  |  |  |
| AK069590.1 | 3-hydroxyisobutyryl-coenzyme A hydrolase | 0.0054 | 1.155 | 123.7±2.4 | 107.1±4.6 |
| AK120964.1 | 4-coumarate-CoA ligase 2 | 0.0003 | 1.624 | 58.5±2.9 | 36.0±1.5 |
| AK064436.1 | ATPase, AAA family protein | 0.0015 | 1.552 | 329.5±22.3 | 212.2±13.6 |
| BI795990 | Chloroplast ATP synthase a chain precursor | 0.0071 | 1.135 | 3656.6±15.0 | 3222.8±147.1 |
| AK063413.1 | CTP synthase family protein | 0.0022 | 1.353 | 249.3±11.3 | 184.3±11.6 |
| **Lipid metabolism** | |  |  |  |  |
| CB659772 | 3-oxoacyl-synthase | 0.0003 | 1.334 | 51.5±1.4 | 38.6±1.3 |
| CR283151 | Ab-hydrolase associated lipase region family protein | 0.0005 | 1.407 | 459.1±12.5 | 326.3±18.7 |
| AK065708.1 | AMP-binding enzyme family protein | 0.0094 | 1.283 | 56.8±4.3 | 44.3±1.7 |
| AK061229.1 | GDSL-like Lipase/Acylhydrolase family protein | 0.0053 | 1.260 | 1339.2±76.0 | 1062.8±41.9 |
| AK121004.1 | Lecithin:cholesterol acyltransferase family protein | 0.0073 | 1.355 | 610.7±40.1 | 450.7±37.8 |
| BX927619 | Lipase family protein | 0.0083 | 1.348 | 126.0±8.8 | 93.5±7.6 |
| AK073298.1 | Lipid phosphate phosphatase 2 | 0.0042 | 1.353 | 414.8±22.6 | 306.5±22.6 |
| AK107901.1 | Nonspecific lipid-transfer protein 2 | 0.0053 | 1.486 | 6064.5±560.8 | 4079.8±277.3 |
| AK108288.1 | Oleosin family protein | 0.0073 | 1.477 | 332.5±27.3 | 225.2±24.9 |
| AK063599.1 | Oleosin family protein | 0.0092 | 1.591 | 1260.1±153.3 | 792.2±78.0 |
| **Nucleotide metabolism** | |  |  |  |  |
| AK065762.1 | 1-imidazole-4-carboxamide isomerase | 0.0026 | 1.276 | 79.6±3.8 | 62.3±2.3 |
| AK101926.1 | Adenosine kinase 2 | 0.0046 | 1.286 | 296.1±19.4 | 230.3±4.6 |
| CR290589 | DNA ligase I,ATP-dependent family protein | 0.0083 | 1.262 | 192.4±13.2 | 152.5±5.3 |
| AK067868.1 | DNA/pantothenate metabolism flavoprotein | 0.0074 | 1.431 | 60.5±5.8 | 42.3±2.5 |
| AK073223.1 | Hydrolase, NUDIX family protein  Linker histone H1 and H5 family protein | 0.0001 | 1.284 | 109.5±1.1 | 85.3±2.1 |
| AK073668.1 |  | 0.0088 | 2.275 | 863.7±175.1 | 379.6±12.8 |
| AK065443.1 | Pseudouridine synthase family protein | 0.0079 | 1.376 | 61.3±2.2 | 44.5±5.4 |
| AK120153.1 | Pyridine nucleotide-disulphide oxidoreductase family protein | 0.0032 | 1.188 | 322.8±13.8 | 271.7±2.1 |
| AB031396.1 | Ribonuclease 2-5A family protein | 0.0052 | 1.251 | 149.9±6.0 | 119.9±7.3 |
| AK111876.1 | Strictosidine synthase family protein | 0.0015 | 1.313 | 1051.4±44.6 | 800.5±33.8 |
| AK063937.1 | TPR Domain containing protein | 0.0036 | 1.107 | 1776.0±43.7 | 1603.6±21.5 |
| AK101363.1 | Type IIB DNA topoisomerase family protein | 0.0078 | 1.172 | 436.1±17.3 | 372.1±14.3 |
| **Organ development** | |  |  |  |  |
| AK102627.1 | CobW protein | 0.0072 | 1.430 | 290.6±14.6 | 203.2±26.2 |
| AK102605.1 | Fb27 | 0.0003 | 1.187 | 488.4±9.9 | 411.5±4.5 |
| AK068711.1 | Fiber protein Fb34 | 0.0073 | 1.145 | 302.1±3.9 | 263.8±12.6 |
| AK067960.1 | MtN3/saliva family protein | 0.004 | 3.028 | 195.4±35.7 | 64.5±13.1 |
| U43931.1 | Oleosin family protein | 0.0019 | 1.144 | 12973.8±177.1 | 11344.6±342.9 |
| AK064375.1 | Protein ULTRAPETALA2 | 0.0018 | 1.397 | 84.2±4.0 | 60.3±4.0 |
| **Photosynthesis** | |  |  |  |  |
| AK119250.1 | Photosystem I P700 chlorophyll a apoprotein A2 | 0.0015 | 1.260 | 61.0±1.5 | 48.4±2.4 |
| AK069447.1 | Transketolase,chloroplast precursor | 0.0031 | 1.522 | 1009.7±86.0 | 663.4±38.2 |
| **Plant hormone biosynthesis** | |  |  |  |  |
| AK102506.1 | AT-hook protein 1 | 0.0064 | 1.304 | 246.2±14.5 | 188.8±12.2 |
| **Protein biosynthesis** | |  |  |  |  |
| AK120520.1 | 40S ribosomal protein S11 | 0.0072 | 1.162 | 3278.5±126.9 | 2820.5±92.2 |
| CF291340 | 40S ribosomal protein S9 | 0.0019 | 1.165 | 373.7±12.1 | 320.7±3.9 |
| AK063884.1 | 50S ribosomal protein L18 | 0.0001 | 1.190 | 851.5±13.7 | 715.5±5.6 |
| AK071249.1 | 60S ribosomal protein L10a-1 | 0.0004 | 1.344 | 4072.7±144.5 | 3031.2±82.8 |
| AK066714.1 | Alanyl-tRNA synthetase | 0.0065 | 1.303 | 56.2±4.0 | 43.1±1.7 |
| AK107052.1 | Aspartyl-tRNA synthetase | 0.0015 | 1.350 | 102.1±4.4 | 75.6±3.9 |
| AK060382.1 | Chloroplast 30S ribosomal protein S3 | 0.0001 | 1.238 | 1044.3±2.7 | 843.6±10.0 |
| AK066521.1 | Lysyl-tRNA synthetase | 0.0039 | 1.245 | 146.1±5.7 | 117.3±6.0 |
| 9632.m00190 | Peptidyl-prolyl cis-trans isomerase 1 | 0.0097 | 1.151 | 1452.7±66.8 | 1261.7±24.7 |
| CB625883 | Phenylalanyl-tRNA synthetase alpha chain | 0.01 | 1.145 | 1117.1±49.4 | 975.8±19.5 |
| CR291564 | Ribosomal protein S11 containing protein | 0.006 | 1.165 | 61.1±2.4 | 52.4±1.4 |
| **Protein degradation** | |  |  |  |  |
| 9630.m04048 | ATP-dependent Clp protease proteolytic subunit | 0.0053 | 1.376 | 77.3±4.1 | 56.2±5.2 |
| CF337464 | Autophagy-related protein 8 precursor | 0.0086 | 1.182 | 127.6±4.0 | 108.0±5.8 |
| AK067739.1 | BPM | 0.0018 | 1.228 | 276.2±4.5 | 224.9±11.2 |
| AK103449.1 | Carboxyl-terminal peptidase | 0.0082 | 1.402 | 66.0±5.5 | 47.1±3.8 |
| AK069442.1 | CUE domain containing protein | 0.0028 | 1.293 | 166.6±5.8 | 128.8±8.1 |
| AK070159.1 | Eukaryotic aspartyl protease family protein | 0.0072 | 1.448 | 62.2±5.5 | 43.0±3.7 |
| AK069151.1 | F-box domain containing protein | 0.0013 | 1.186 | 277.9±2.7 | 234.2±9.0 |
| AK105430.1 | F-box domain containing protein | 0.0033 | 1.206 | 78.3±3.0 | 64.9±2.2 |
| AK071522.1 | F-box domain containing protein | 0.0012 | 1.220 | 120.3±4.4 | 98.6±1.3 |
| AK101235.1 | F-box domain containing protein | 0.0047 | 1.299 | 354.1±20.6 | 272.6±13.8 |
| AK071294.1 | F-box domain containing protein | 0.0053 | 1.305 | 76.3±5.4 | 58.5±1.5 |
| AK060772.1 | F-box domain containing protein | 0.0076 | 1.345 | 630.8±21.3 | 469.0±52.0 |
| AK068467.1 | F-box protein interaction domain containing protein | 0.003 | 1.282 | 91.8±4.5 | 71.7±3.0 |
| AK065062.1 | GDA1/CD39 family protein | 0.0021 | 1.503 | 278.3±17.7 | 185.3±14.5 |
| AK058912.1 | Glycine cleavage system H protein,mitochondrial precursor | 0.0077 | 1.370 | 825.6±49.8 | 602.4±59.8 |
| AK103626.1 | GPI transamidase component PIG-S | 0.0053 | 1.137 | 346.7±6.5 | 305.0±11.4 |
| AK101289.1 | Huntingtin interacting protein K | 0.0023 | 1.131 | 1560.3±44.3 | 1379.9±9.3 |
| AY151043.1 | MCB1 protein | 0.0022 | 1.321 | 292.9±14.4 | 221.8±10.1 |
| AU071158 | Molybdopterin converting factor,subunit 1 family protein | 0.0042 | 1.418 | 181.5±14.9 | 128.0±5.0 |
| AK068775.1 | OTU-like cysteine protease family protein | 0.0034 | 1.407 | 68.6±3.4 | 48.8±4.4 |
| AK100611.1 | PDZ domain family protein | 0.0043 | 1.640 | 157.8±16.5 | 96.2±7.9 |
| AK066996.1 | Peptidase family M1 containing protein | 0.0017 | 1.173 | 1503.9±40.4 | 1282.1±32.0 |
| AK110647.1 | Peptidase family M20/M25/M40 containing protein | 0.0062 | 1.521 | 1491.5±142.7 | 980.5±88.4 |
| AK059215.1 | Peptidase family M41 containing protein | 0.0028 | 1.411 | 78.9±2.5 | 55.9±5.6 |
| AK070316.1 | Prolyl oligopeptidase family protein | 0.0085 | 1.445 | 287.0±27.2 | 198.7±16.3 |
| AK101083.1 | Prolyl oligopeptidase family protein | 0.0015 | 1.530 | 188.1±11.9 | 123.0±8.4 |
| AK073569.1 | Protease inhibitor/seed storage/LTP family protein | 0.0085 | 1.254 | 212.1±15.0 | 169.1±3.5 |
| AK067175.1 | SAND family protein | 0.0085 | 1.206 | 191.3±10.1 | 158.7±5.9 |
| AK069785.1 | Serine carboxypeptidase family protein | 0.0006 | 1.297 | 96.4±3.8 | 74.3±0.9 |
| AK064814.1 | Serine carboxypeptidase family protein | 0.0071 | 1.767 | 58.0±8.3 | 32.8±2.1 |
| AK121368.1 | Ubiquinol-cytochrome c reductase complex 14 kDa protein | 0.0046 | 1.192 | 884.0±30.9 | 741.5±29.9 |
| AK103230.1 | Ubiquitin carboxyl-terminal hydrolase 14 | 0.0033 | 1.151 | 845.1±23.7 | 734.0±19.7 |
| AK122086.1 | WD-repeat protein 8 | 0.0092 | 1.258 | 293.7±18.0 | 233.5±12.9 |
| AK105806.1 | Xylanase inhibitor TAXI-IV | 0.01 | 1.752 | 891.1±118.6 | 508.8±81.2 |
| AF344453.1 | YDG/SRA domain containing protein | 0.005 | 1.165 | 526.8±6.1 | 452.2±22.3 |
| **Protein folding and transport** | |  |  |  |  |
| AK071240.1 | 17.5 kDa class II heat shock protein | 0.008 | 1.475 | 7168.0±771.1 | 4860.0±266.6 |
| AK066738.1 | Cation transporter/ATPase,N-terminus family protein | 0.0062 | 1.178 | 176.0±5.3 | 149.5±6.9 |
| AK105364.1 | Chaperone protein dnaJ 10 | 0.0071 | 1.140 | 850.2±30.5 | 746.1±18.2 |
| AK066545.1 | Endomembrane protein 70 containing protein | 0.0071 | 1.186 | 300.3±13.0 | 253.2±9.4 |
| AK102032.1 | Hydrolase, alpha/beta fold family protein | 0.0008 | 2.174 | 1157.4±102.3 | 532.4±56.1 |
| AK102204.1 | MATE efflux family protein | 0.0095 | 1.731 | 57.8±8.7 | 33.4±2.5 |
| AK067917.1 | Membrane associated protein | 0.0055 | 1.352 | 763.5±53.7 | 564.9±33.0 |
| AK121722.1 | PDR5-like ABC transporter | 0.0003 | 1.290 | 53.3±1.2 | 41.3±1.4 |
| **Redox homeostasis** | |  |  |  |  |
| AK068919.1 | 2-cys peroxiredoxin BAS1 | 0.0100 | 1.241 | 1208.3±51.5 | 974.0±33.5 |
| AK060849.1 | Acyl-coenzyme A oxidase 1.2, peroxisomal | 0.0014 | 1.289 | 1063.7±51.5 | 825.0±11.6 |
| AK108458.1 | Ascorbate peroxidase 4 | 0.0100 | 1.329 | 81.3±4.2 | 61.2±2.8 |
| AK070842.1 | Aascorbate peroxidase 4 | 0.0100 | 1.222 | 1027.5±30.6 | 840.9±13.7 |
| CA762987 | ATP-NAD kinase family protein | 0.0067 | 1.335 | 143.5±9.2 | 107.5±7.9 |
| AK059818.1 | Glutathione S-transferase GSTF2 | 0.0073 | 1.270 | 5563.7±124.7 | 4379.8±388.0 |
| AK069604.1 | Glutathione S-transferase GSTU1 | 0.0098 | 1.329 | 694.1±3.3 | 522.2±64.2 |
| BI812695 | Glyoxalase family protein | 0.0038 | 1.380 | 1925.7±115.3 | 1395.4±98.9 |
| AK065317.1 | Isoflavone reductase homolog | 0.0028 | 1.669 | 68.1±7.0 | 40.8±1.8 |
| AK102459.1 | Monodehydroascorbate reductase | 0.0001 | 2.477 | 748.8±29.8 | 302.3±45.7 |
| AK099848.1 | Monodehydroascorbate reductase | 0.0100 | 1.262 | 467.4±40.7 | 370.5±23.5 |
| 9634.m03728 | NAD dependent epimerase/dehydratase family protein | 0.0056 | 1.425 | 52.1±3.1 | 36.6±3.8 |
| AK107947.1 | NAD dependent epimerase/dehydratase family protein | 0.0077 | 1.476 | 144.8±15.8 | 98.1±4.2 |
| AK071324.1 | NADH-cytochrome b5 reductase | 0.0034 | 1.329 | 384.3±11.4 | 289.2±23.9 |
| AK105359.1 | Oxidoreductase, short chain dehydrogenase/reductase family protein | 0.0004 | 1.958 | 101.8±3.2 | 52.0±7.0 |
| AK121818.1 | Oxidoreductase,2OG-Fe oxygenase family protein | 0.0012 | 1.320 | 573.0±25.5 | 434.1±15.1 |
| AK068314.1 | Oxidoreductase,2OG-Fe oxygenase family protein | 0.0094 | 1.537 | 198.1±16.9 | 128.9±19.2 |
| AK105776.1 | Oxidoreductase,short chain dehydrogenase/reductase family protein | 0.0086 | 1.311 | 601.8±51.2 | 459.1±5.1 |
| AK067136.1 | Oxidoreductase,zinc-binding dehydrogenase family protein | 0.009 | 1.233 | 1352.2±77.4 | 1096.8±51.7 |
| AK069374.1 | Oxidoreductase,zinc-binding dehydrogenase family protein | 0.0035 | 1.239 | 1227.0±53.4 | 990.5±39.6 |
| AK073849.1 | Oxidoreductase,zinc-binding dehydrogenase family protein | 0.0017 | 1.345 | 167.4±9.4 | 124.5±3.1 |
| 9632.m03698 | Peroxidase 65 precursor | 0.0062 | 1.509 | 253.3±16.4 | 167.9±22.7 |
| AK058456.1 | Phospholipid hydroperoxide glutathione peroxidase | 0.0100 | 1.448 | 1585.8±94.6 | 1095.2±58.2 |
| AK070522.1 | RRM-containing protein | 0.0074 | 1.345 | 91.9±1.8 | 68.3±7.9 |
| D85239.1 | Superoxide dismutase,chloroplast precursor | 0.0004 | 1.673 | 3540.1±204.9 | 2116.0±108.2 |
| AK065111.1 | Thiamine pyrophosphate enzyme,central domain containing protein | 0.0069 | 1.174 | 652.4±32.3 | 555.6±5.0 |
| AK102564.1 | Thioredoxin-like 8,chloroplast precursor | 0.0008 | 1.130 | 265.3±4.7 | 234.8±3.3 |
| **RNA processing** | |  |  |  |  |
| AK063379.1 | Methyltransferase, FkbM family protein | 0.0076 | 1.169 | 120.0±1.2 | 102.7±5.9 |
| AK102566.1 | Metalloenzyme superfamily protein | 0.0091 | 1.246 | 1157.2±80.1 | 928.4±24.8 |
| **Signal transduction or/TF** | |  |  |  |  |
| AK106057.1 | AP2 domain containing protein | 0.0028 | 1.379 | 58.5±1.0 | 42.4±4.1 |
| AK119342.1 | Armadillo/beta-catenin-like repeat family protein | 0.0026 | 1.202 | 91.7±2.4 | 76.3±3.2 |
| AK101241.1 | Armadillo/beta-catenin-like repeat family protein | 0.0076 | 1.645 | 174.2±22.2 | 105.9±8.5 |
| AK069003.1 | ATPase, histidine kinase, DNA gyrase B- and HSP90-like domain containing protein | 0.0096 | 1.633 | 146.4±20.2 | 89.7±6.1 |
| AY371050.1 | ATPase, histidine kinase, DNA gyrase B- and HSP90-like domain containing protein | 0.0092 | 1.325 | 68.2±6.0 | 51.5±1.5 |
| AK071455.1 | Auxin response factor 8 | 0.004 | 1.519 | 119.3±7.6 | 78.5±9.1 |
| AK101780.1 | Auxin-regulated protein | 0.0084 | 1.204 | 104.5±5.1 | 86.7±3.7 |
| AK101356.1 | B3 DNA binding domain containing protein | 0.0011 | 1.514 | 92.2±4.2 | 60.9±4.9 |
| AK106748.1 | BES1/BZR1 homolog protein 1 | 0.0015 | 1.469 | 104.6±3.8 | 71.2±6.4 |
| CB630069 | BTB/POZ domain containing protein | 0.0025 | 1.215 | 109.5±4.7 | 90.2±1.4 |
| AK067168.1 | BTB/POZ domain-containing protein, putative | 0.0058 | 1.176 | 523.5±13.0 | 445.0±21.6 |
| AK064971.1 | C2 domain-containing protein | 0.001 | 1.582 | 71.7±4.3 | 45.3±3.2 |
| AK058544.1 | Calcium-binding EF hand family protein | 0.0074 | 1.313 | 232.7±19.1 | 177.2±1.6 |
| AK071566.1 | Coiled-coil domain containing 12 | 0.0044 | 1.581 | 215.9±19.2 | 136.6±14.0 |
| AK067978.1 | CRAL/TRIO,N-terminus family protein | 0.0049 | 1.367 | 213.8±14.1 | 156.4±10.6 |
| 9639.m03658 | Cyclin-dependent kinase inhibitor family protein | 0.0089 | 1.590 | 276.4±7.2 | 173.8±36.6 |
| AK060709.1 | Cytokinin receptor CRE1b | 0.0043 | 1.300 | 76.4±4.3 | 58.7±3.0 |
| AK103165.1 | DHHC zinc finger domain containing protein | 0.0056 | 1.140 | 175.9±5.5 | 154.3±4.2 |
| AK073653.1 | DHHC zinc finger domain containing protein | 0.0014 | 1.963 | 156.8±15.8 | 79.9±6.0 |
| C23582 | Dof domain, zinc finger family protein | 0.0035 | 1.358 | 76.9±4.3 | 56.6±3.8 |
| AK105920.1 | EF hand family protein | 0.0033 | 1.893 | 63.4±7.7 | 33.5±3.0 |
| 9635.m00711 | Exostosin family protein, putative | 0.01 | 1.176 | 68.9±3.3 | 58.6±2.0 |
| AK101652.1 | FK506-binding protein 4 | 0.0036 | 1.364 | 1173.6±72.5 | 860.7±50.8 |
| AK073946.1 | G-patch domain containing protein | 0.0071 | 1.139 | 388.3±10.9 | 341.0±11.9 |
| AK106487.1 | GRF zinc finger family protein | 0.0094 | 1.588 | 58.7±7.1 | 37.0±3.8 |
| AK121135.1 | Histidine kinase 2 | 0.0033 | 1.116 | 147.3±2.9 | 132.0±3.1 |
| AK103607.1 | Histone-like transcription factor and archaeal histone family protein | 0.0085 | 1.162 | 70.7±2.6 | 60.8±2.4 |
| AF145728.1 | Homeobox associated leucine zipper family protein | 0.0014 | 1.447 | 263.7±13.4 | 182.3±12.0 |
| AB101647.1 | Homeobox protein OCL1 | 0.0032 | 1.278 | 161.3±6.1 | 126.2±7.4 |
| AY581257.1 | Hpt domain containing protein | 0.002 | 1.915 | 298.4±30.0 | 155.8±17.3 |
| AK101159.1 | Hus1 protein | 0.0091 | 1.126 | 131.8±4.1 | 117.0±3.5 |
| AK062441.1 | IQ calmodulin-binding motif family protein | 0.0026 | 1.163 | 289.9±10.0 | 249.1±3.5 |
| AK069365.1 | Kelch motif family protein | 0.0073 | 1.263 | 62.6±3.7 | 49.6±2.5 |
| AK072805.1 | Kelch motif family protein | 0.005 | 1.303 | 790.3±18.2 | 606.7±53.9 |
| AK065064.1 | Kelch repeat-containing serine/threonine phosphoesterase family protein | 0.0095 | 1.211 | 170.3±10.3 | 140.6±4.0 |
| AK072520.1 | LIM domain containing protein | 0.0045 | 1.250 | 49.8±1.2 | 39.9±2.7 |
| AK060067.1 | Low molecular weight phosphotyrosine protein phosphatase containing protein | 0.0055 | 1.407 | 473.2±34.0 | 336.4±27.1 |
| AK072434.1 | Maf family protein | 0.0001 | 1.229 | 156.6±0.0 | 127.5±3.4 |
| AK101674.1 | Myb30 | 0.0022 | 1.544 | 92.8±1.2 | 60.1±8.1 |
| AK062250.1 | Myb-like DNA-binding domain | 0.0079 | 1.348 | 57.5±4.6 | 42.7±2.4 |
| AB064519.1 | Myb-like DNA-binding domain containing protein | 0.0034 | 2.116 | 74.8±11.0 | 35.4±1.0 |
| AK069869.1 | MYB-like protein 2, putative | 0.0089 | 1.271 | 108.9±1.4 | 85.7±8.3 |
| AK070561.1 | Myosin-like protein | 0.0001 | 2.610 | 850.7±35.0 | 326.0±23.1 |
| AK106305.1 | PHD finger transcription factor | 0.0008 | 1.325 | 157.9±4.3 | 119.2±6.1 |
| AK065628.1 | Phosphatidylinositol 3- and 4-kinase family protein | 0.0071 | 1.229 | 412.9±23.5 | 336.0±11.5 |
| AK120080.1 | Phosphatidylinositol-4-phosphate 5-Kinase family protein | 0.0014 | 1.459 | 108.7±5.5 | 74.5±5.1 |
| AK122015.1 | Phospholipase D.Active site motif family protein | 0.004 | 1.411 | 51.1±3.8 | 36.2±2.0 |
| AK066883.1 | Protein kinase domain containing protein | 0.0011 | 1.273 | 92.0±2.1 | 72.2±3.5 |
| AK059352.1 | Protein kinase domain containing protein | 0.0021 | 1.458 | 303.5±11.8 | 208.2±20.1 |
| AK102480.1 | Protein kinase domain containing protein | 0.0059 | 1.517 | 55.6±3.8 | 36.6±4.8 |
| AK064032.1 | Protein kinase family protein | 0.0014 | 1.362 | 146.4±5.4 | 107.4±6.6 |
| AK105501.1 | Protein kinase family protein | 0.0037 | 1.540 | 169.7±12.7 | 110.2±11.3 |
| AK069274.1 | Protein phosphatase 2C | 0.0017 | 1.522 | 160.3±10.1 | 105.3±7.6 |
| AK106529.1 | Protein prenyltransferase alpha subunit repeat containing protein | 0.0031 | 1.423 | 154.2±10.3 | 108.4±7.1 |
| AK073894.1 | Receptor family ligand binding region containing protein | 0.0043 | 1.245 | 53.0±2.6 | 42.6±1.7 |
| AK101339.1 | Receptor protein kinase CRINKLY4 precursor | 0.0032 | 1.543 | 66.0±6.2 | 42.8±1.4 |
| AK107793.1 | Serine/threonine protein phosphatase | 0.0021 | 1.109 | 145.1±2.5 | 130.8±2.5 |
| AK067377.1 | SGT1 protein | 0.0085 | 1.409 | 409.3±38.5 | 290.6±18.2 |
| 9630.m01398 | Sodium/calcium exchanger protein | 0.0093 | 1.200 | 53.8±1.7 | 44.8±2.9 |
| AK059374.1 | TAK14 | 0.0054 | 1.136 | 54.8±0.8 | 48.2±1.9 |
| AK120648.1 | Two-component response regulator ARR12 | 0.0082 | 1.728 | 134.8±19.9 | 78.0±3.6 |
| AK121352.1 | Yippee-like protein At4g27740 | 0.0013 | 1.280 | 403.8±13.8 | 315.5±12.9 |
| AK101299.1 | YT521-B-like family protein | 0.0093 | 1.119 | 1368.1±43.2 | 1222.6±31.6 |
| AK071014.1 | Zinc finger family protein | 0.0093 | 1.316 | 108.9±9.4 | 82.7±2.1 |
| AK064407.1 | Zinc finger, C3HC4 type family protein | 0.0063 | 1.161 | 393.0±11.9 | 338.5±13.4 |
| AK109529.1 | Zinc finger, C3HC4 type family protein | 0.0028 | 1.426 | 265.6±18.4 | 186.3±10.2 |
| AK069562.1 | Zinc finger, C3HC4 type family protein | 0.0089 | 1.464 | 169.7±12.6 | 115.9±15.0 |
| AK069097.1 | Zinc finger,C2H2 type family protein | 0.0024 | 1.415 | 792.2±50.2 | 559.9±30.1 |
| AK073266.1 | Zinc finger,C3HC4 type family protein | 0.0018 | 1.461 | 4636.3±235.2 | 3172.4±251.0 |
| AB126086.1 | ZIP zinc/iron transport family protein | 0.0065 | 1.799 | 116.4±15.0 | 64.7±8.5 |
| **Substance transport** | |  |  |  |  |
| AF022737.1 | Aquaporin RWC3 | 0.006 | 1.368 | 2314.2±186.7 | 1692.3±77.2 |
| AK072421.1 | Boron transporter-like protein 2 | 0.0006 | 1.648 | 333.8±20.9 | 202.5±10.8 |
| AK103471.1 | Brittle-1 protein, chloroplast precursor | 0.0032 | 1.162 | 77.0±1.0 | 66.3±2.8 |
| AK107381.1 | Chemocyanin precursor | 0.0021 | 1.437 | 1826.2±68.2 | 1271.2±117.6 |
| AK102185.1 | Glycosyl hydrolases family 17 protein | 0.0082 | 2.103 | 233.0±43.1 | 110.8±5.4 |
| AK067646.1 | Mitochondrial carrier protein | 0.01 | 1.133 | 112.1±1.7 | 99.0±4.6 |
| AK072060.1 | Mitochondrial carrier protein | 0.0064 | 1.302 | 116.0±8.9 | 89.1±0.6 |
| AK101966.1 | Mitochondrial import inner membrane translocase subunit Tim17/Tim22/Tim23 family protein | 0.0028 | 1.251 | 407.7±17.2 | 325.9±13.0 |
| AK066636.1 | Pectate lyase family protein | 0.0097 | 1.339 | 68.2±4.7 | 50.9±4.4 |
| AK068351.1 | POT family protein | 0.0006 | 1.460 | 58.9±3.1 | 40.3±1.2 |
| AK072123.1 | Potassium transporter 11 | 0.0087 | 1.278 | 242.4±0.8 | 189.8±19.0 |
| AK065464.1 | Potassium transporter 11 | 0.0011 | 1.327 | 125.9±2.0 | 94.9±6.0 |
| **Transcription** | |  |  |  |  |
| AK100660.1 | Cleavage and polyadenylation specificity factor,73 kDa subunit | 0.0012 | 1.180 | 744.6±22.9 | 631.3±6.0 |
| AK106405.1 | Cleavage stimulation factor,50 kDa subunit | 0.002 | 1.181 | 205.8±1.6 | 174.3±7.4 |
| AK105308.1 | DNA-directed RNA polymerase alpha chain | 0.0036 | 1.283 | 501.3±28.8 | 390.8±12.0 |
| X15901.1 | DNA-directed RNA polymerase beta chain | 0.0056 | 1.367 | 62.4±4.8 | 45.6±2.4 |
| AK100529.1 | Endonuclease III-like protein 1 | 0.0034 | 1.190 | 77.1±1.0 | 64.8±3.3 |
| AK073113.1 | Exosome complex exonuclease RRP40 | 0.0097 | 1.187 | 149.4±1.2 | 125.9±8.7 |
| AK120902.1 | Helix-loop-helix DNA-binding domain containing protein | 0.0004 | 1.337 | 178.6±4.2 | 133.6±5.6 |
| AK064663.1 | HMG box family protein | 0.0035 | 1.218 | 513.0±8.4 | 421.3±24.3 |
| AK061675.1 | Piwi domain containing protein | 0.0022 | 1.229 | 2544.7±115.8 | 2070.0±19.5 |
| AK065274.1 | Piwi domain containing protein | 0.0027 | 1.337 | 706.1±42.1 | 528.3±19.5 |
| AB047689.2 | PolI-like DNA polymerase | 0.0015 | 1.266 | 140.1±4.7 | 110.6±4.6 |
| AK073417.1 | Pre-mRNA splicing factor ATP-dependent RNA helicase PRP16 | 0.0023 | 1.207 | 471.6±19.1 | 390.8±6.6 |
| AK066304.1 | PWWP domain containing protein | 0.007 | 1.336 | 490.6±23.2 | 367.2±34.9 |
| 9630.m04803 | RNA polymerase II 15.9 kDa subunit | 0.0096 | 1.138 | 413.3±15.1 | 363.2±11.0 |
| AK102518.1 | RNA recognition motif family protein | 0.0042 | 1.220 | 431.4±16.9 | 353.6±15.6 |
| AK059444.1 | RNA recognition motif family protein | 0.0084 | 1.262 | 173.6±12.6 | 137.6±2.5 |
| 9632.m03244 | RNA recognition motif family protein | 0.008 | 1.358 | 65.6±4.7 | 48.3±3.9 |
| 9632.m02251 | RNA recognition motif family protein | 0.003 | 1.521 | 131.8±10.0 | 86.7±6.9 |
| CA761882 | RNA recognition motif family protein | 0.0007 | 4.074 | 54.5±7.1 | 13.4±2.4 |
| AK103702.1 | RNA-binding protein | 0.0023 | 1.119 | 296.3±7.4 | 264.7±2.8 |
| AK120075.1 | RNase3 domain containing protein | 0.0002 | 1.279 | 158.2±3.7 | 123.7±2.5 |
| AK073431.1 | SAM domain family protein | 0.0049 | 1.779 | 462.1±59.1 | 259.8±19.6 |
| AB101645.1 | START domain containing protein | 0.0066 | 1.446 | 153.5±8.9 | 106.1±13.1 |
| D87261.1 | TCP family transcription factor containing protein | 0.0021 | 1.399 | 142.3±6.4 | 101.7±7.5 |
| AK069714.1 | Transcription factor | 0.0015 | 1.332 | 300.1±13.5 | 225.4±10.0 |
| **Unknown pathway** | |  |  |  |  |
| AK062427.1 | Enzyme of the cupin superfamily | 0.0001 | 1.451 | 2824.0±43.8 | 1946.9±49.8 |
| AK107293.1 | La domain-containing protein | 0.0007 | 1.183 | 107.9±2.8 | 91.2±1.0 |
| CA759213 | Phytanoyl-CoA dioxygenase | 0.0056 | 1.260 | 3048.2±172.5 | 2419.0±102.4 |
| AK069474.1 | PQ loop repeat family protein | 0.0001 | 1.468 | 81.8±2.0 | 55.7±2.0 |
| AY323487.1 | Retrotransposon | 0.0026 | 1.292 | 109.9±6.1 | 85.1±2.0 |
| AK120701.1 | Retrotransposon protein,Ty3-gypsy subclass | 0.0078 | 1.678 | 491.0±55.0 | 292.7±42.6 |
| CF313824 | Transposon protein | 0.0074 | 1.114 | 138.5±4.7 | 124.3±1.2 |
| AK120083.1 | Transposon protein | 0.0047 | 1.225 | 103.0±2.9 | 84.0±5.0 |
| AK101478.1 | Transposon protein | 0.0045 | 1.263 | 433.4±18.5 | 343.2±19.7 |
| AK066317.1 | Transposon protein | 0.0078 | 1.271 | 631.0±42.3 | 496.7±20.7 |
| AK120918.1 | Transposon protein | 0.0047 | 1.363 | 81.7±3.3 | 60.0±5.7 |
| AK069462.1 | Transposon protein | 0.0063 | 1.449 | 164.8±15.3 | 113.7±7.2 |
| AK064531.1 | Transposon protein | 0.0072 | 1.647 | 260.2±31.9 | 158.0±14.3 |
| AK070418.1 | Transposon protein | 0.0098 | 1.675 | 79.3±7.8 | 47.3±9.0 |
|  |  |  |  |  |  |
| ***Down-regulated genes*** | |  |  |  |  |
| **Amide acid metabolism** | |  |  |  |  |
| BI805246 | Agmatine deiminase | 0.0084 | 0.784 | 451.7±36.1 | 576.0±25.9 |
| AK106883.1 | Amino acid permease | 0.0001 | 0.886 | 703.7±4.2 | 793.9±9.2 |
| AK064140.1 | Aminotransferase | 0.0021 | 0.715 | 208.5±14.5 | 291.5±14.1 |
| AK073549.1 | Aminotransferase | 0.01 | 0.650 | 1754.6±255.0 | 2698.4±246.5 |
| AK065212.1 | Aminotransferase ACS12 | 0.0007 | 0.706 | 501.0±35.5 | 709.2±14.2 |
| AK104978.1 | Aminotransferase, class IV family protein | 0.0034 | 0.634 | 113.7±17.2 | 179.5±6.1 |
| AK071368.1 | GCN5-related N-acetyltransferase | 0.0008 | 0.730 | 717.7±46.1 | 982.8±21.3 |
| AK065301.1 | Glutamate dehydrogenase | 0.0076 | 0.711 | 74.6±10.3 | 104.8±2.0 |
| AK064826.1 | Glutamate-1-semialdehyde 2,1-aminomutase,chloroplast precursor | 0.0011 | 0.724 | 223.6±12.7 | 308.9±11.9 |
| AK121856.1 | Patellin-5 | 0.0055 | 0.706 | 1293.0±161.7 | 1832.2±57.3 |
| **Carbohydrate metabolism** | |  |  |  |  |
| AK065370.1 | ADP-ribosylation factor | 0.0099 | 0.803 | 1121.7±85.1 | 1396.9±58.7 |
| 9635.m00643 | ALG6, ALG8 glycosyltransferase family protein | 0.009 | 0.765 | 180.6±14.2 | 236.1±14.4 |
| NM_191752.1 | Alpha amylase,catalytic domain containing protein | 0.0076 | 0.554 | 281.8±75.7 | 508.8±22.9 |
| AY187621.1 | Beta-fructofuranosidase | 0.0033 | 0.544 | 173.5±37.6 | 319.2±14.1 |
| BE040411 | Beta-galactosidase precursor | 0.0003 | 0.587 | 346.0±12.8 | 589.7±34.1 |
| AK102715.1 | Beta-galactosidase precursor | 0.0021 | 0.696 | 625.5±58.9 | 899.3±31.6 |
| AK072356.1 | Cellulose synthase A catalytic subunit 3 | 0.0089 | 0.777 | 1249.9±127.4 | 1608.2±27.8 |
| AK100475.1 | Cellulose synthase A catalytic subunit 8 | 0.0018 | 0.786 | 176.1±10.8 | 224.0±3.1 |
| D29697.1 | Chalcone synthase | 0.0049 | 0.815 | 243.4±16.1 | 298.6±5.4 |
| AK102152.1 | D-mannose binding lectin family protein | 0.0038 | 0.853 | 177.2±7.5 | 207.7±4.4 |
| AK107370.1 | D-mannose binding lectin family protein | 0.0066 | 0.621 | 1825.2±317.3 | 2937.8±194.8 |
| AK111065.1 | Eukaryotic-type carbonic anhydrase family protein | 0.0034 | 0.564 | 187.1±37.4 | 331.7±15.1 |
| AK068247.1 | Glucan endo-1,3-beta-glucosidase GII precursor | 0.0047 | 0.535 | 39.9±7.6 | 74.6±7.3 |
| AB164463.1 | Glycogenin | 0.0026 | 0.707 | 50.7±4.7 | 71.7±2.8 |
| 9635.m04746 | Glycosyl hydrolase family 14 protein | 0.001 | 0.646 | 86.9±3.9 | 134.6±8.9 |
| AK073110.1 | Glycosyl hydrolase family 3 N terminal domain containing protein | 0.0013 | 0.514 | 309.9±55.8 | 603.5±28.5 |
| AK105267.1 | Glycosyl transferase,group 1 family protein | 0.0026 | 0.623 | 391.1±56.0 | 627.6±25.6 |
| AK070014.1 | Glycosyltransferase | 0.0002 | 0.714 | 257.3±10.6 | 360.5±9.3 |
| AK070134.1 | Glycosyltransferase family 14 protein | 0.0097 | 0.734 | 464.4±48.9 | 632.9±39.3 |
| AK064011.1 | Hexokinase 1 | 0.0057 | 0.794 | 468.9±35.5 | 590.8±16.5 |
| 9639.m02588 | Hexose transporter | 0.0061 | 0.867 | 83.4±2.1 | 96.2±3.6 |
| AK072296.1 | Inositol polyphosphate kinase family protein | 0.0093 | 0.748 | 803.6±30.7 | 1073.9±94.6 |
| AK063126.1 | Lichenase II precursor | 0.0018 | 0.548 | 1474.0±227.7 | 2687.8±173.4 |
| AK102748.1 | Membrane-anchored endo-1,4-beta-glucanase | 0.0025 | 0.695 | 1714.4±188.8 | 2467.7±41.6 |
| NM_183526.1 | Phosphoenolpyruvate carboxylase 4 | 0.0036 | 0.790 | 350.2±5.6 | 443.3±25.7 |
| 9639.m00344 | Phosphoglycerate mutase family protein | 0.009 | 0.577 | 113.2±14.1 | 196.2±26.7 |
| AK065644.1 | Phosphoglycerate mutase family protein | 0.0014 | 0.671 | 443.3±12.6 | 660.5±45.8 |
| AK073897.1 | Phosphoribosylanthranilate transferase | 0.0002 | 0.827 | 1228.2±7.7 | 1484.5±33.0 |
| AK063871.1 | UDP-glucose 4-epimerase GEPI48 | 0.0085 | 0.740 | 393.7±34.6 | 531.7±35.6 |
| AU108098 | Xylanase inhibitor protein | 0.0005 | 0.849 | 228.2±4.5 | 269.0±5.0 |
| AK064180.1 | Xylanase inhibitor protein | 0.0086 | 0.670 | 1943.3±179.74 | 2899.9±294.9 |
| **Cell cycle/biogenesis** | |  |  |  |  |
| AK070020.1 | Agenet domain containing protein | 0.0093 | 0.629 | 190.0±37.9 | 302.3±16.5 |
| AK073842.1 | Beta-Ig-H3 domain-containing protein | 0.0007 | 0.679 | 1291.5±44.9 | 1901.5±100.4 |
| 9632.m03709 | Cell division protein FtsH, putative | 0.0036 | 0.882 | 56.1±0.9 | 63.6±1.9 |
| AK121992.1 | CENP-E like kinetochore protein | 0.0059 | 0.653 | 347.4±48.4 | 532.1±35.2 |
| AK061100.1 | Cyclin,N-terminal domain containing protein | 0.0057 | 0.425 | 153.1±53.9 | 360.6±39.4 |
| AK063070.1 | Exo70 exocyst complex subunit family protein | 0.0057 | 0.713 | 44.4±2.8 | 62.3±5.0 |
| AK061447.1 | MSP domain containing protein | 0.0058 | 0.604 | 351.8±71.5 | 582.1±19.7 |
| AK107407.1 | No apical meristem | 0.0059 | 0.843 | 7057.2±193.8 | 8368.2±377.2 |
| BI802765 | No apical meristem | 0.0089 | 0.701 | 3638.1±476.6 | 5191.6±304.1 |
| BI802765 | No apical meristem protein | 0.0007 | 0.805 | 4692.2±102.0 | 5826.0±183.5 |
| 9639.m02843 | No apical meristem protein | 0.0077 | 0.512 | 192.2±53.7 | 375.5±34.7 |
| AK107407.1 | No apical meristem, putative | 0.004 | 0.830 | 5756.0±218.2 | 6931.0±263.5 |
| AK059397.1 | Seed maturation protein PM23 | 0.0002 | 0.746 | 2119.9±69.4 | 2842.4±56.5 |
| AK060393.1 | Stomatin-like protein 2 | 0.0041 | 0.750 | 403.3±23.4 | 537.5±18.1 |
| **Cell rescue/defense** | |  |  |  |  |
| AK073727.1 | ABA-induced protein | 0.001 | 0.631 | 1412.5±83.1 | 2238.3±142.4 |
| AK103909.1 | Alkylated DNA repair protein alkB homolog | 0.0036 | 0.783 | 129.8±5.8 | 165.7±8.4 |
| AF247162.1 | Alpha-expansin 15 precursor | 0.0026 | 0.593 | 246.4±10.7 | 415.6±42.3 |
| AK119834.1 | Alpha-expansin 3 precursor | 0.0001 | 0.408 | 43.4±2.3 | 106.4±4.4 |
| AK105833.1 | BAG domain-containing protein | 0.0087 | 0.687 | 621.3±96.4 | 904.7±34.5 |
| AK106735.1 | Caffeoyl-CoA O-methyltransferase 2 | 0.0002 | 0.455 | 27.9±1.7 | 61.3±4.1 |
| X89891.1 | Caleosin 2 | 0.005 | 0.716 | 2469.9±149.6 | 3447.7±262.5 |
| AK070347.1 | Caleosin related protein | 0.0023 | 0.693 | 1386.6±130.0 | 2002.0±83.9 |
| AK120195.1 | callose synthase | 0.0085 | 0.668 | 136.1±9.4 | 203.6±22.3 |
| AK111651.1 | CPR5 protein | 0.0038 | 0.655 | 1261.0±156.7 | 1924.9±108.9 |
| AK121175.1 | Cytochrome P450 family protein | 0.01 | 0.592 | 32.63±4.9 | 55.1±6.9 |
| AK107961.1 | DnaJ domain containing protein | 0.0003 | 0.519 | 120.4±15.9 | 232.2±4.4 |
| 9634.m01819 | Embryogenesis transmembrane protein | 0.0097 | 0.781 | 59.2±4.7 | 75.8±4.1 |
| AK065800.1 | FHA domain containing protein | 0.0001 | 0.591 | 738.9±43.6 | 1249.7±16.1 |
| AK068772.1 | Glycosyl hydrolase family 1 protein | 0.0009 | 0.429 | 92.3±14.7 | 215.4±18.7 |
| NM_193731.1 | Glycosyl hydrolase family 1 protein | 0.0078 | 0.723 | 2042.9±179.1 | 2826.2±207.9 |
| CB966906 | Glycosyl hydrolase family 1 protein, expressed | 0.0025 | 0.761 | 38.5±2.6 | 50.5±1.7 |
| AK110722.1 | Haemolysin-III related family protein | 0.008 | 0.860 | 2100.6±37.8 | 2442.7±114.5 |
| AK122005.1 | Harpin-induced protein 1 containing protein | 0.0044 | 0.709 | 132.6±6.9 | 186.9±14.7 |
| AK069019.1 | Harpin-induced protein 1 containing protein | 0.0068 | 0.602 | 462.2±58.2 | 767.2±84.9 |
| AK065454.1 | Heavy metal-induced putative protein 1 | 0.0026 | 0.637 | 862.5±74.1 | 1354.1±104.1 |
| 9629.m00808 | Holocarboxylase synthetase | 0.0087 | 0.795 | 542.1±42.5 | 681.9±27.4 |
| BI797790 | Hypoxia induced protein conserved region containing protein | 0.0062 | 0.788 | 4589.7±383.4 | 5824.9±133.9 |
| CF327155 | Hypoxia induced protein conserved region containing protein | 0.0058 | 0.801 | 4622.7±346.2 | 5771.1±132.3 |
| AF085174.3 | Immutans protein | 0.0053 | 0.630 | 54.0±4.6 | 85.8±8.9 |
| 9633.m00699 | Leucine Rich Repeat family protein | 0.0041 | 0.739 | 101.7±9.5 | 137.6±4.6 |
| AK109613.1 | Leucine Rich Repeat family protein | 0.0037 | 0.835 | 503.8±24.6 | 603.0±14.1 |
| AK071870.1 | Leucine Rich Repeat family protein | 0.0001 | 0.638 | 556.4±30.7 | 872.4±14.8 |
| AF140722.2 | Multiple stress-responsive zinc-finger protein ISAP1 | 0.001 | 0.592 | 831.0±105.3 | 1403.2±49.4 |
| 9640.m02341 | Negatively light-regulated protein | 0.0096 | 0.685 | 55.9±5.5 | 81.7±7.9 |
| CA764876 | Oxysterol-binding protein | 0.0038 | 0.460 | 253.8±61.0 | 551.8±60.0 |
| 9629.m03448 | Pentatricopeptide | 0.0099 | 0.774 | 65.8±3.7 | 84.9±6.2 |
| AK071786.1 | Pentatricopeptide, putative | 0.0042 | 0.745 | 127.7±12.0 | 171.4±4.7 |
| CF319324 | Protein SUR2 | 0.0092 | 0.747 | 184.3±5.9 | 246.7±22.1 |
| AK060379.1 | Remorin, C-terminal region family protein | 0.0005 | 0.521 | 256.3±19.2 | 491.6±35.5 |
| AK060392.1 | Remorin,C-terminal region family protein | 0.0009 | 0.535 | 250.5±13.4 | 468.2±40.5 |
| AK070317.1 | SCP-like extracellular protein | 0.002 | 0.599 | 86.1±12.9 | 143.7±5.1 |
| AK072643.1 | Syntaxin 51 | 0.0097 | 0.811 | 1212.9±92.3 | 1494.7±50.1 |
| AK063768.1 | UV excision repair protein Rad23 containing protein | 0.0008 | 0.675 | 1567.6±124.0 | 2322.4±71.3 |
| CB631702 | Wound-responsive family protein | 0.0014 | 0.757 | 432.1±23.7 | 570.7±18.9 |
| **Energy pathway** | |  |  |  |  |
| AK058604.1 | Acid phosphatase 1 precursor | 0.0054 | 0.532 | 639.8±43.0 | 1203.7±173.4 |
| AK066142.1 | ATPase, AAA family protein | 0.0083 | 0.867 | 1731.5±91.2 | 1997.2±25.2 |
| AK099459.1 | Vacuolar ATP synthase 16 kDa proteolipid subunit | 0.0017 | 0.801 | 1374.4±1.3 | 1715.3±79.2 |
| **Lipid metabolism** | |  |  |  |  |
| AK071888.1 | 3-oxoacyl-synthase I,chloroplast precursor | 0.0033 | 0.513 | 169.2±15.9 | 329.9±41.3 |
| AK070093.1 | CDP-diacylglycerol-glycerol-3-phosphate 3-phosphatidyltransferase family protein | 0.0047 | 0.807 | 1237.7±67.5 | 1534.5±60.1 |
| AK062676.1 | Esterase | 0.0023 | 0.825 | 141.4±5.5 | 171.4±5.1 |
| AK101968.1 | Fatty acid desaturase family protein | 0.0079 | 0.643 | 263.5±50.7 | 409.6±8.5 |
| AK103085.1 | Fatty acid hydroxylase family protein | 0.0069 | 0.741 | 1265.3±112.4 | 1707.1±98.3 |
| AK100992.1 | GDSL-like Lipase/Acylhydrolase family protein | 0.0006 | 0.621 | 102.8±6.4 | 165.5±8.8 |
| AK073443.1 | GDSL-like Lipase/Acylhydrolase family protein | 0.0041 | 0.611 | 1706.35±110.4 | 2793.2±298.5 |
| AK100170.1 | Glycerophosphoryl diester phosphodiesterase 1 precursor | 0.006 | 0.595 | 472.7±101.9 | 794.0±24.6 |
| AK108161.1 | GNS1/SUR4 membrane family protein, putative | 0.0094 | 0.796 | 131.1±7.1 | 164.6±10.2 |
| NM_187882.1 | Lipase class 3 family protein | 0.0061 | 0.729 | 175.8±9.9 | 241.1±18.9 |
| AK067101.1 | Lipase class 3 family protein | 0.0097 | 0.714 | 481.5±37.5 | 674.3±61.4 |
| AK064554.1 | Lipase family protein | 0.008 | 0.769 | 479.2±38.4 | 622.9±33.1 |
| AK109152.1 | MBOAT family protein | 0.0039 | 0.649 | 269.1±26.7 | 414.5±32.3 |
| AB112060.1 | Monogalactosyldiacylglycerol synthase family protein | 0.003 | 0.576 | 812.1±81.2 | 1410.9±139.0 |
| AK071777.1 | Phosphatidate cytidylyltransferase | 0.0068 | 0.751 | 256.0±12.1 | 341.0±26.0 |
| **Nucleotide metabolism** | |  |  |  |  |
| AK069606.1 | Adenine phosphoribosyltransferase 2 | 0.0001 | 0.606 | 213.7±7.0 | 352.5±4.2 |
| AK066688.1 | Adenylate kinase,chloroplast | 0.0077 | 0.781 | 1374.2±91.5 | 1760.3±99.1 |
| CB670040 | Adenylate kinase,chloroplast precursor | 0.0045 | 0.816 | 576.9±32.0 | 706.6±22.5 |
| 9629.m04767 | FAD-binding domain-containing protein | 0.0013 | 0.516 | 70.6±10.7 | 136.9±9.7 |
| AK066193.1 | Phosphoadenosine phosphosulfate | 0.0077 | 0.686 | 215.9±28.0 | 314.6±20.2 |
| AK064269.1 | Polyadenylate-binding protein 2 | 0.0019 | 0.364 | 401.0±153.2 | 1101.4±68.5 |
| AK067210.1 | Polyadenylate-binding protein 2 | 0.0002 | 0.559 | 1615.0±64.8 | 2889.2±159.9 |
| AK067210.1 | Polyadenylate-binding protein 2 | 0.0003 | 0.570 | 2264.1±152.6 | 3974.8±213.1 |
| AK119900.1 | Rhodanese-like domain containing protein | 0.0082 | 0.713 | 2515.6±315.4 | 3526.5±172.4 |
| AK119900.1 | Rhodanese-like domain containing protein | 0.0097 | 0.702 | 2631.9±396.6 | 3747.4±126.4 |
| **Organ development** | |  |  |  |  |
| AK121884.1 | COBRA-like protein 2 precursor | 0.0013 | 0.586 | 887.8±103.4 | 1516.1±87.8 |
| NM_193604.1 | LOB domain protein 22 | 0.0047 | 0.610 | 147.5±17.8 | 241.8±22.4 |
| **Photosynthesis** | |  |  |  |  |
| NM_197133.1 | Chloroplast inner envelope protein | 0.0013 | 0.718 | 1922.1±140.0 | 2678.5±85.0 |
| **Protein biosynthesis** | |  |  |  |  |
| AK066987.1 | CRS1/YhbY domain containing protein | 0.0044 | 0.650 | 52.9±7.8 | 81.5±3.5 |
| AK059693.1 | Peptide transporter PTR2 | 0.0097 | 0.820 | 802.4±43.4 | 978.5±49.3 |
| AK058219.1 | Protein translation factor SUI1 homolog | 0.0083 | 0.811 | 3571.8±123.8 | 4404.4±270.2 |
| M23743.1 | Rice mRNA for prolamine | 0.0017 | 0.778 | 1695.6±86.3 | 2179.4±70.8 |
| AK066713.1 | Tyrosyl-tRNA synthetase family protein | 0.0001 | 0.779 | 333.9±5.8 | 428.9±9.1 |
| **Protein degradation** | |  |  |  |  |
| AK072930.1 | F-box domain containing protein | 0.0016 | 0.575 | 45.7±6.9 | 79.5±3.3 |
| 9636.m03607 | F-box domain containing protein | 0.0071 | 0.502 | 75.1±12.9 | 149.7±22.0 |
| AK105235.1 | F-box domain containing protein | 0.0039 | 0.720 | 124.2±9.4 | 172.5±10.3 |
| 9636.m03608 | F-box domain containing protein | 0.0001 | 0.396 | 50.9±2.9 | 128.6±8.0 |
| 9636.m02917 | F-box domain containing protein | 0.0003 | 0.864 | 145.4±1.3 | 168.3±3.1 |
| 9629.m07064 | F-box domain containing protein | 0.0081 | 0.643 | 152.9±20.6 | 237.9±22.0 |
| AK121479.1 | F-box domain containing protein | 0.005 | 0.579 | 230.7±8.2 | 398.1±51.1 |
| AK107242.1 | F-box domain containing protein | 0.0065 | 0.663 | 310.6±15.4 | 468.6±50.2 |
| AK105235.1 | F-box domain containing protein | 0.0068 | 0.678 | 330.5±41.3 | 487.8±33.1 |
| AK065651.1 | F-box domain containing protein | 0.0063 | 0.628 | 56.8±4.0 | 90.5±10.3 |
| AK101481.1 | F-box domain containing protein | 0.0009 | 0.572 | 744.5±63.7 | 1301.7±87.7 |
| AK060317.1 | F-box domain containing protein | 0.0034 | 0.727 | 2452.9±184.6 | 3375.6±180.0 |
| AK066566.1 | Metalloendopeptidase | 0.0099 | 0.707 | 2521.8±377.6 | 3567.7±105.8 |
| 9636.m03120 | Metallopeptidase family M24 containing protein | 0.0083 | 0.840 | 198.5±8.2 | 236.4±10.8 |
| AK099464.1 | Pyrrolidone-carboxylate peptidase family protein | 0.0011 | 0.768 | 3652.7±161.0 | 4755.2±159.5 |
| AK102160.1 | Ubiquitin carboxyl-terminal hydrolase 1 | 0.0001 | 0.138 | 9.8±1.9 | 70.7±2.5 |
| AK069639.1 | Ubiquitin carboxyl-terminal hydrolase family protein | 0.0047 | 0.819 | 2498.0±152.0 | 3049.6±72.2 |
| AK073821.1 | Ubiquitin-conjugating enzyme E2 M | 0.0088 | 0.831 | 4907.2±121.5 | 5907.2±341.3 |
| AK100714.1 | Ubiquitin-conjugating enzyme family protein | 0.0017 | 0.771 | 238.7±9.3 | 309.6±13.6 |
| AK069668.1 | Ubiquitin-like protein SMT3 | 0.0034 | 0.765 | 154.0±5.2 | 201.4±12.1 |
| AK100423.1 | U-box domain containing protein | 0.0035 | 0.200 | 77.5±13.3 | 387.8±86.1 |
| AK062592.1 | U-box domain containing protein | 0.0017 | 0.629 | 793.7±83.8 | 1262.6±67.9 |
| NM_185608.1 | U-box domain containing protein | 0.0062 | 0.788 | 1438.1±125.7 | 1825.3±20.3 |
| **Protein folding and transport** | |  |  |  |  |
| NM_189889.1 | ABC transporter family protein | 0.0086 | 0.680 | 236.0±18.9 | 347.1±35.3 |
| AK103938.1 | Aquaporin PIP2.4 | 0.0002 | 0.889 | 5474.2±67.1 | 6157.8±58.8 |
| AK072840.1 | Endomembrane protein 70 containing protein | 0.0058 | 0.845 | 745.0±21.7 | 967.9±69.6 |
| AK121320.1 | Endomembrane protein 70 containing protein | 0.002 | 0.769 | 877.0±41.3 | 1140.8±48.0 |
| 9631.m00540 | Hsp20/alpha crystallin family protein | 0.0014 | 0.475 | 82.8±13.5 | 174.4±14.7 |
| AK109873.1 | MATE efflux family protein | 0.0058 | 0.758 | 208.3±10.3 | 274.8±18.8 |
| 9632.m02891 | MATE efflux family protein, expressed | 0.0001 | 0.767 | 49.5±1.2 | 64.6±1.4 |
| AK072910.1 | Membrane protein | 0.0034 | 0.759 | 89.1±4.9 | 117.4±6.1 |
| AK059988.1 | Membrane protein | 0.0092 | 0.809 | 2815.0±219.0 | 3481.4±108.4 |
| 9631.m05200 | PRA1 family protein | 0.0058 | 0.818 | 506.5±29.5 | 619.5±21.4 |
| AK060006.1 | PRA1 family protein | 0.0014 | 0.847 | 1374.7±34.1 | 1622.5±43.1 |
| AK059702.1 | Protein transport protein | 0.0084 | 0.778 | 2031.4±160.9 | 2611.8±131.6 |
| **Redox homeostasis** | |  |  |  |  |
| AK071203.1 | 12-oxophytodienoate reductase 3 | 0.007 | 0.704 | 167.5±10.9 | 237.9±21.3 |
| AK066324.1 | Cation exchanger | 0.0001 | 0.013 | 3.7±0.3 | 294.5±20.2 |
| AK103187.1 | Cytochrome oxidase assembly protein | 0.0017 | 0.640 | 287.4±21.9 | 449.1±30.4 |
| AK120232.1 | Ferredoxin-3,chloroplast precursor | 0.0063 | 0.696 | 839.5±42.4 | 1206.8±113.5 |
| 9631.m04316 | Glutaredoxin family protein | 0.002 | 0.670 | 89.1±10.2 | 133.0±3.1 |
| AK067828.1 | Glutaredoxin family protein | 0.0046 | 0.733 | 842.6±85.3 | 1149.6±37.2 |
| 9632.m03516 | Lipoxygenase 5 | 0.0079 | 0.847 | 101.4±4.7 | 119.7±4.4 |
| AK100965.1 | NAD dependent epimerase/dehydratase family protein | 0.0049 | 0.433 | 42.0±4.0 | 97.0±16.5 |
| 9633.m04087 | Oxidoreductase, 2OG-Fe oxygenase family protein | 0.0003 | 0.770 | 40.8±1.8 | 53.0±0.4 |
| AK058935.1 | Oxidoreductase,2OG-Fe oxygenase family protein | 0.0079 | 0.723 | 565.7±33.4 | 782.3±68.4 |
| CB666869 | Peroxidase family protein | 0.0031 | 0.842 | 107.2±5.3 | 127.4±1.4 |
| AK068639.1 | Peroxisome assembly protein 10 | 0.0047 | 0.651 | 185.1±20.4 | 284.5±22.4 |
| AK122041.1 | Protein disulfide isomerase | 0.005 | 0.629 | 427.6±68.4 | 679.5±37.1 |
| **RNA processing** | |  |  |  |  |
| CB650564 | ATP-dependent RNA helicase DBP2 | 0.0047 | 0.610 | 476.8±89.9 | 781.8±22.5 |
| AK062123.1 | ATP-dependent RNA helicase DBP2 | 0.0049 | 0.803 | 1746.0±128.7 | 2174.0±28.0 |
| AK067560.1 | S3 self-incompatibility locus-linked pollen 3.15 protein, | 0.007 | 0.761 | 1188.0±73.2 | 1560.3±103.1 |
| **Signal transduction or/TF** | |  |  |  |  |
| AK121051.1 | 39 kDa EF-Hand containing protein | 0.0009 | 0.568 | 1326.3±73.9 | 2335.5±181.7 |
| AY377427.1 | AN1-like Zinc finger family protein | 0.0003 | 0.823 | 1281.0±34.2 | 1556.7±24.2 |
| AK108758.1 | Ankyrin repeat domain protein 2 | 0.0067 | 0.469 | 39.9±3.8 | 85.2±14.7 |
| AK066651.1 | Anther ethylene-upregulated protein ER1 | 0.0047 | 0.751 | 483.7±36.9 | 644.5±32.2 |
| AY341827.1 | AP2 domain containing protein | 0.0019 | 0.544 | 91.0±17.3 | 167.2±5.0 |
| 9636.m04399 | AP2 domain containing protein | 0.0013 | 0.485 | 275.5±56.4 | 567.9±27.9 |
| 9632.m05041 | AP2 domain containing protein | 0.0033 | 0.564 | 1439.9±297.4 | 2552.9±76.2 |
| 9634.m02249 | Auxin-responsive protein IAA8 | 0.0019 | 0.777 | 176.1±7.3 | 226.5±9.6 |
| AK061535.1 | BTB/POZ domain containing protein | 0.0027 | 0.765 | 141.0±7.2 | 184.2±8.8 |
| U04295.1 | BZIP transcription factor family protein | 0.002 | 0.670 | 337.7±34.2 | 503.8±21.3 |
| AK105466.1 | C2 domain-containing protein | 0.0001 | 0.142 | 40.4±8.8 | 284.7±28.6 |
| AK121215.1 | Calcium-binding EF hand family protein | 0.0006 | 0.807 | 1590.5±9.6 | 1970.3±66.7 |
| AB042550.1 | Calcium-dependent protein kinase,isoform 11 | 0.0034 | 0.771 | 407.5±11.2 | 528.9±31.8 |
| AF048691.1 | Calcium-dependent protein kinase,isoform 11 | 0.0053 | 0.649 | 1613.1±140.9 | 2486.1±234.9 |
| AK103306.1 | Calcium-dependent protein kinase,isoform AK1 | 0.0067 | 0.336 | 88.0±19.2 | 261.5±55.1 |
| NM_197303.1 | CDPK-related protein kinase | 0.0008 | 0.629 | 340.8±31.9 | 541.7±21.1 |
| AK120297.1 | CHY zinc finger family protein | 0.0017 | 0.547 | 268.2±51.5 | 490.4±4.4 |
| AK059604.1 | CRAL/TRIO, N-terminus family protein | 0.0035 | 0.710 | 1494.0±45.5 | 2105.1±164.8 |
| AK068782.1 | EF hand family protein | 0.01 | 0.709 | 165.9±24.9 | 233.8±5.7 |
| AK065151.1 | EF hand family protein | 0.0044 | 0.483 | 409.6±76.7 | 848.0±106.3 |
| AK109541.1 | EF hand family protein | 0.0058 | 0.594 | 455.1±46.9 | 766.4±88.6 |
| AK073335.1 | Exostosin family protein | 0.0007 | 0.741 | 1005.6±17.3 | 1356.5±62.0 |
| AK071529.1 | GTP-binding protein | 0.0057 | 0.791 | 385.2±10.7 | 486.7±30.8 |
| 9630.m04210 | GTP-binding protein YPTM2 | 0.0058 | 0.789 | 489.6±42.1 | 620.2±1.3 |
| NM_190466.1 | IQ calmodulin-binding motif family protein | 0.0087 | 0.457 | 101.0±29.3 | 221.1±32.1 |
| AK120454.1 | LSD1 zinc finger family protein | 0.0021 | 0.785 | 41.6±2.6 | 53.1±1.1 |
| AK065964.1 | Matrixin family protein | 0.0034 | 0.771 | 113.1±6.4 | 146.6±6.8 |
| AK072172.1 | Mitogen-activated protein kinase | 0.0076 | 0.845 | 92.7±4.9 | 109.7±3.2 |
| AK100023.1 | Mitogen-activated protein kinase 1 | 0.0026 | 0.831 | 573.8±27.3 | 690.9±13.3 |
| AK066520.1 | Myb-like DNA-binding domain containing protein | 0.0012 | 0.794 | 441.4±13.9 | 555.9±20.0 |
| BX898176 | Myb-like DNA-binding domain containing protein | 0.0001 | 0.797 | 965.4±24.1 | 1210.7±8.2 |
| AK068138.1 | MYB-like transcription factor DIVARICATA | 0.0076 | 0.598 | 127.0±25.1 | 212.2±15.9 |
| AK068552.1 | Nodulation receptor kinase precursor | 0.008 | 0.546 | 76.1±5.9 | 139.4±21.5 |
| AK101685.1 | Nuclear transport factor 2 domain containing protein | 0.0006 | 0.749 | 520.6±29.4 | 694.7±9.6 |
| AK066262.1 | Nucleoside-triphosphatase, putative | 0.0028 | 0.799 | 625.6±25.9 | 783.0±32.7 |
| AK106955.1 | Protein kinase APK1B, chloroplast precursor | 0.0047 | 0.349 | 108.5±12.3 | 310.5±60.3 |
| AK100479.1 | Protein kinase APK1B,chloroplast precursor | 0.0065 | 0.858 | 389.9±19.9 | 454.2±8.0 |
| AK071585.1 | Protein kinase domain containing protein | 0.0045 | 0.546 | 63.4±6.5 | 116.2±14.5 |
| AK105946.1 | Protein kinase domain containing protein | 0.0054 | 0.475 | 75.1±25.6 | 158.0±5.3 |
| AK111766.1 | Protein kinase domain containing protein | 0.0019 | 0.479 | 174.7±31.3 | 365.0±32.6 |
| AK111746.1 | Protein kinase domain containing protein | 0.0097 | 0.670 | 184.2±32.7 | 275.0±9.2 |
| AK101120.1 | Protein kinase domain containing protein | 0.0063 | 0.733 | 451.1±53.9 | 615.0±3.6 |
| AK066731.1 | RabGAP/TBC domain-containing protein | 0.0015 | 0.747 | 651.9±24.5 | 872.3±42.6 |
| AK071461.1 | Ras-related protein Rab11B | 0.0056 | 0.656 | 198.6±21.1 | 302.7±25.7 |
| AK059799.1 | Ras-related protein Rab-2-B | 0.0044 | 0.777 | 2440.3±58.2 | 3139.1±200.6 |
| AK070320.1 | Ras-related protein Rab7 | 0.0015 | 0.737 | 1497.7±59.3 | 2032.2±104.5 |
| AK107384.1 | Response regulator receiver domain containing protein | 0.0001 | 0.272 | 33.7±9.1 | 123.9±4.0 |
| 9634.m01062 | Scarecrow 3 protein | 0.003 | 0.674 | 155.0±10.2 | 230.1±17.3 |
| AK121649.1 | Ser/Thr protein phosphatase family protein | 0.0001 | 0.301 | 80.8±3.8 | 268.8±18.6 |
| AK107301.1 | Ser/Thr protein phosphatase family protein | 0.0047 | 0.592 | 1243.6±221.7 | 2101.0±138.1 |
| AK120921.1 | Serine/threonine-protein kinase RLCKVII | 0.001 | 0.878 | 396.0±6.0 | 450.8±9.1 |
| AK071154.1 | Small GTP-binding protein domain containing protein | 0.0094 | 0.772 | 204.2±8.4 | 264.5±20.7 |
| AK121106.1 | Sphingosine kinase | 0.0057 | 0.673 | 551.1±67.6 | 819.4±53.6 |
| NM_188761.1 | TAZ zinc finger family protein | 0.0088 | 0.662 | 187.4±23.7 | 283.1±25.3 |
| AK059734.1 | Two-component response regulator ARR3 | 0.0022 | 0.383 | 161.9±14.2 | 422.4±62.9 |
| AK102968.1 | WD domains,G-beta repeats containing protein | 0.0063 | 0.863 | 1953.5±97.3 | 2264.4±33.3 |
| AK100995.1 | XYPPX repeat family protein | 0.0016 | 0.676 | 971.1±57.7 | 1437.1±88.8 |
| AK065106.1 | zinc finger family protein | 0.0006 | 0.715 | 703.1±49.1 | 983.0±9.2 |
| 9635.m03804 | Zinc finger protein | 0.0026 | 0.686 | 200.6±14.2 | 292.3±19.1 |
| AK119276.1 | Zinc finger, C2H2 type family protein | 0.0026 | 0.617 | 46.7±6.2 | 75.7±4.2 |
| AY077725.1 | Zinc finger, C2H2 type family protein | 0.0089 | 0.730 | 2133.0±248.2 | 2921.7±143.3 |
| AK105338.1 | Zinc finger, C3HC4 type family protein | 0.0036 | 0.685 | 184.9±15.4 | 270.1±18.6 |
| AY395294.1 | Zinc finger,C2H2 type family protein | 0.0027 | 0.533 | 129.4±27.7 | 242.6±10.6 |
| 9630.m05845 | Zinc finger,C3HC4 type family protein | 0.01 | 0.888 | 50.1±0.4 | 56.4±2.3 |
| AK106311.1 | Zinc finger,C3HC4 type family protein | 0.0068 | 0.588 | 548.1±114.5 | 931.5±59.6 |
| AK067751.1 | Zinc finger,C3HC4 type family protein | 0.0055 | 0.720 | 552.6±45.6 | 767.6±50.7 |
| AK067053.1 | Zinc finger,C3HC4 type family protein | 0.002 | 0.596 | 807.7±122.9 | 1354.8±47.1 |
| AK120485.1 | Zinc finger,C3HC4 type family protein | 0.0016 | 0.688 | 1158.3±112.6 | 1683.7±38.9 |
| AK064311.1 | Zinc finger,C3HC4 type family protein | 0.0055 | 0.663 | 1248.8±132.6 | 1883.6±151.7 |
| AK070908.1 | Zinc finger,ZZ type family protein | 0.0004 | 0.597 | 1235.9±128.8 | 2070.4±39.3 |
| AF332876.1 | Zinc-finger protein 1 | 0.0082 | 0.745 | 137.7±10.6 | 184.7±12.9 |
| **Substance transport** | |  |  |  |  |
| 9631.m05485 | Glycosyl hydrolases family 17 protein | 0.0034 | 0.527 | 179.0±20.4 | 339.9±39.7 |
| 9631.m05485 | Glycosyl hydrolases family 17 protein | 0.0037 | 0.458 | 408.5±45.7 | 891.0±130.2 |
| AK059357.1 | Major Facilitator Superfamily protein | 0.003 | 0.836 | 158.4±7.8 | 189.5±3.0 |
| 9631.m00726 | Mitochondrial carrier | 0.0039 | 0.709 | 1672.1±139.8 | 2358.8±140.9 |
| AK109109.1 | Phosphate transporter family protein | 0.0082 | 0.840 | 59.6±1.1 | 70.9±3.9 |
| 9639.m01654 | POT family protein | 0.0003 | 0.631 | 1254.5±100.8 | 1987.5±26.5 |
| 9639.m01654 | POT family protein | 0.0007 | 0.637 | 1316.2±132.6 | 2065.1±28.9 |
| AK105438.1 | Transmembrane protein 49 | 0.0027 | 0.647 | 347.8±31.1 | 537.8±38.5 |
| **Transcription** | |  |  |  |  |
| CA762859 | CCAAT-binding transcription factor subunit B family protein | 0.0003 | 0.785 | 1357.0±50.4 | 1727.7±20.7 |
| CB658384 | DNA-binding protein RAV1 | 0.0006 | 0.576 | 672.2±28.4 | 1167.0±81.1 |
| AK072725.1 | Folate/biopterin transporter family protein | 0.0001 | 0.420 | 110.0±6.2 | 261.5±14.0 |
| 9629.m07118 | GRAS family transcription factor containing protein | 0.0012 | 0.567 | 202.8±20.4 | 357.7±25.6 |
| AK058386.1 | Helicase SEN1 | 0.0008 | 0.653 | 1075.0±87.1 | 1645.6±67.0 |
| AK070076.1 | Helicase SEN1, putative | 0.0037 | 0.660 | 577.5±72.4 | 875.7±44.9 |
| AK058386.1 | Helicase SEN1, putative | 0.0035 | 0.686 | 1227.9±110.4 | 1789.7±112.1 |
| 9634.m00622 | Helix-loop-helix DNA-binding domain containing protein | 0.0063 | 0.524 | 110.5±31.5 | 210.7±9.8 |
| AY222337.1 | Helix-loop-helix DNA-binding domain containing protein | 0.0009 | 0.772 | 421.5±20.9 | 545.8±11.9 |
| CB622390 | Initiator-binding protein | 0.0071 | 0.830 | 228.1±13.9 | 274.9±7.8 |
| AK058507.1 | Pollen-specific protein SF3 | 0.0005 | 0.503 | 2204.7±264.3 | 4379.6±243.9 |
| 9639.m03552 | RNA recognition motif family protein | 0.0032 | 0.663 | 65.1±3.4 | 98.3±8.4 |
| CB618243 | RNA recognition motif family protein | 0.0058 | 0.836 | 151.6±9.5 | 181.3±1.2 |
| AK106338.1 | RNA recognition motif family protein | 0.0015 | 0.562 | 609.4±39.4 | 1083.5±98.0 |
| AK059172.1 | RNA recognition motif family protein | 0.0014 | 0.865 | 3142.1±74.3 | 3632.8±79.2 |
| 9630.m04012 | RNase H domain-containing protein | 0.002 | 0.319 | 37.5±4.4 | 117.6±18.8 |
| AK070198.1 | Splicing factor U2AF 35 kDa subunit | 0.007 | 0.867 | 2250.9±101.3 | 2594.9±58.7 |
| 9631.m02180 | Transcription factor RF2b | 0.0021 | 0.585 | 181.5±28.4 | 310.3±13.1 |
| AY466471.1 | Transcription factor RF2b | 0.0001 | 0.705 | 818.1±11.7 | 1160.6±38.2 |
| **Unknown pathway** | |  |  |  |  |
| AK073162.1 | Actin-depolymerizing factor 6 | 0.004 | 0.815 | 4689.1±143.9 | 5753.0±274.4 |
| AK100454.1 | Ataxin-2 C-terminal region family protein | 0.0018 | 0.845 | 5616.2±129.0 | 6647.0±206.3 |
| AK106511.1 | Bicoid-interacting protein 3 containing protein | 0.0004 | 0.739 | 89.8±0.7 | 121.5±5.0 |
| AK103217.1 | DIP2 protein | 0.0031 | 0.815 | 1191.5±49.5 | 1461.5±54.2] |
| AK069774.1 | Integral to membrane protein | 0.0067 | 0.851 | 3907.8±48.1 | 4594.4±225.6 |
| NM_195253.1 | PAP/25A associated domain containing protein | 0.0058 | 0.641 | 87.1±12.7 | 135.8±9.3 |
| AK121448.1 | Retrotransposon protein | 0.0001 | 0.060 | 28.9±4.8 | 484.5±56.0 |
| AK073899.1 | Retrotransposon protein | 0.0038 | 0.894 | 49.5±1.2 | 55.4±1.2 |
| AK119423.1 | Retrotransposon protein | 0.0021 | 0.737 | 108.7±4.9 | 147.4±8.1 |
| AK109781.1 | Retrotransposon protein | 0.0026 | 0.380 | 139.4±30.2 | 366.7±50.5 |
| AK068969.1 | Retrotransposon protein | 0.0033 | 0.641 | 35.5±3.2 | 55.5±4.5 |
| NM_194888.1 | Retrotransposon protein | 0.0014 | 0.684 | 730.0±38.2 | 1067.8±64.0 |
| AK108333.1 | Retrotransposon protein,Ty1-copia subclass | 0.004 | 0.641 | 183.8±29.2 | 286.9±6.7 |
| AK109781.1 | Retrotransposon protein,Ty3-gypsy subclass | 0.0015 | 0.342 | 128.4±16.9 | 375.1±52.2 |
| AK110492.1 | Transposon protein | 0.0008 | 0.771 | 69.3±3.0 | 89.9±2.6 |
| NM_196179.1 | Transposon protein | 0.0096 | 0.620 | 79.3±17.8 | 127.8±3.3 |
| NM_195718.1 | Transposon protein | 0.0075 | 0.582 | 83.6±16.7 | 143.7±12.4 |
| AK120573.1 | Transposon protein | 0.0087 | 0.846 | 157.2±4.6 | 185.8±9.2 |
| AK062359.1 | Transposon protein,CACTA,En/Spm sub-class | 0.0071 | 0.707 | 350.3±44.7 | 495.4±21.0 |

^1^ Functional classification of 623 differentially expressed genes between CSSL50-1 and Asominori as detected by one-way ANOVA (P value <0.01).
