# Supplementary material for: Transcriptome analysis of grain-filling caryopses reveals involvement of multiple regulatory pathways in chalky grain formation in rice
Source: BMC Genomics. 2010 Dec 30;11:730. doi: 10.1186/1471-2164-11-730 (PMC3023816; doi:10.1186/1471-2164-11-730)
Supplement: Additional file 5 — Sequence of primers used for RT-PCR. A list of primers used for RT-PCR and the sequence information. [file 1471-2164-11-730-S5.DOC]

**Additional file 5**

**Table S3:** Sequence of primers used for RT-PCR.^1^

| **NCBI No** | **Encoded protein** | **Cycles** |  | **Sequence (5'-to-3')** |
| --- | --- | --- | --- | --- |
| AK058456.1 | Peroxidase | 30 | F: | ACTGCACCCATCTACAAGTTTCTG |
|  |  |  | R: | GTCAAAACTTGCTCATCGCAGTCG |
| AK058912.1 | Glycine cleavage system H protein | 30 | F: | CCGTGCTGAAGGATCTGAAGTATGC |
|  |  |  | R: | TCAAGGCGCATTCTTTGCAGATCAC |
| AK070561.1 | Myosin-like protein | 30 | F: | ACCGCATACCTCGTGAATAC |
|  |  |  | R: | GCCATTGGTGGTCCATAAGC |
| AK070705.1 | Phosphoglycerate kinase | 27 | F: | GAAAAAGCCAAGTCAAAGGGAGTC |
|  |  |  | R: | TGCTTGCCACCAGTCTTATGCGTC |
| AK071249.1 | 60S ribosomal protein | 27 | F: | TCCTCGTCTCCTTGGTCCTGGTCTC |
|  |  |  | R: | GCAGCATAACACACATAGAATTTCC |
| AK072765.1 | Heparanase-like protein | 30 | F: | GCAGGAAACAGCAGCAAGGAATCAC |
|  |  |  | R: | GCGTAATACTCAACAAGAAGGCAAG |
| AK073266.1 | Zinc finger,C3HC4 type family protein | 27 | F: | AGTTGGTACGGAAAGCAGAGG |
|  |  |  | R: | TACATGGGCGACAATGAGCG |
| AK101015.1 | HEAT repeat family protein | 28 | F: | TCAATGTGCTTCAGCAAGAGCGTCC |
|  |  |  | R: | ATGTAGAGAAAGAACAACCATAACC |
| AK101652.1 | FK506-binding protein | 30 | F: | GCAAGCCAGAATACGCATACG |
|  |  |  | R: | TCAAACAGGAGCAACAACGC |
| AK102032.1 | Hydrolase | 30 | F: | CTCTTCCCTGCCTCCAGCGTCATCG |
|  |  |  | R: | AACGAAGCACCAAGAAACGCACTCC |
| AK102459.1 | Monodehydroascorbate reductase | 30 | F: | CGGAGGTGGCTACATAGGAC |
|  |  |  | R: | CGGGAGTAGAAGTAGGGCAG |
| AK107494.1 | Glucose-6-phosphate isomerase | 30 | F: | GATCGCTGCCACTGTCCTGAAGAG |
|  |  |  | R: | GGAAGCACTTACTTTACAAATCGG |
| BI812695 | Glyoxalase | 29 | F: | CGCCACCACCTCGCCTTCTCCGTCG |
|  |  |  | R: | AGTGCAATCCAGTAACATATAGAGC |
| CR282303 | Starch debranching enzyme | 28 | F: | ACAAGAAAGAAGAATCCTCTGACT |
|  |  |  | R: | TACTATGCTATACTTTGTTCTTTC |
| AK063871.1 | UDP-glucose 4-epimerase | 30 | F: | GGGAGGAGACCGTCACTTAC |
|  |  |  | R: | CCATTCTGCTTGGTGGAGTC |
| AK071368.1 | GCN5-related N-acetyltransferase | 30 | F: | CATTAAAGAGGCGATGTAGCAAG |
|  |  |  | R: | TTTCCAAATCACAAGGACCAG |
| AK072356.1 | Cellulose synthase | 29 | F: | AGAGGAGGGTGTTGAAGGTG |
|  |  |  | R: | GAGGAGACCCTGGAAGACGG |
| AK103085.1 | Fatty acid hydroxylase family protein | 29 | F: | GTCTGCTGGTTGCTTGTG |
|  |  |  | R: | GTGGCTCAGTTGTTCTTTCC |
| AK121884.1 | COBRA-like protein 2 precursor | 30 | F: | GCCAGAATAATAAACCAGGAAGC |
|  |  |  | R: | CGACGGTTTATGGTTAGGTATCTG |
| NM_191752.1 | Alpha amylase | 30 | F: | ATAATCAAGATGCCCACAGG |
|  |  |  | R: | TACGATGACACTTCCCAGAC |

^1^A list of primers used for RT-PCR and the sequence information.
